# Phytochemical Characterization of *Rhus coriaria* L. Extracts by Headspace Solid-Phase Micro Extraction Gas Chromatography, Comprehensive Two-Dimensional Liquid Chromatography, and Antioxidant Activity Evaluation

**DOI:** 10.3390/molecules27051727

**Published:** 2022-03-07

**Authors:** Katia Arena, Emanuela Trovato, Francesco Cacciola, Ludovica Spagnuolo, Elisa Pannucci, Paolo Guarnaccia, Luca Santi, Paola Dugo, Luigi Mondello, Laura Dugo

**Affiliations:** 1 Department of Chemical, Biological, Pharmaceutical and Environmental Sciences, University of Messina, 98168 Messina, Italy; arenak@unime.it (K.A.); emanuela.trovato1@unime.it (E.T.); pdugo@unime.it (P.D.); lmondello@unime.it (L.M.); 2 Department of Biomedical, Dental, Morphological and Functional Imaging Sciences, University of Messina, 98125 Messina, Italy; 3 Department of Sciences and Technologies for Human and Environment, University Campus Bio-Medico of Rome, 00128 Rome, Italy; l.spagnuolo@unicampus.it (L.S.); e.pannucci@unicampus.it (E.P.); l.dugo@unicampus.it (L.D.); 4 Department of Agriculture, Food Science and Environment (Di3A), University of Catania, 95124 Catania, Italy; paolo.guarnaccia@unict.it; 5 Department of Agriculture and Forest Sciences (DAFNE), University of Tuscia, 01100 Viterbo, Italy; luca.santi@unitus.it; 6 Chromaleont s.r.l., c/o Department of Chemical, Biological, Pharmaceutical and Environmental Sciences, University of Messina, 98168 Messina, Italy

**Keywords:** *Rhus coriaria* L., sumac, volatiles, polyphenols, LC×LC, GC, mass spectrometry, antioxidant activity

## Abstract

*Rhus coriaria* L. (Anacardiaceae), commonly known as sumac, has been used since ancient times for many different applications, and nowadays is used mostly as a spice obtained from its in the Mediterranean and the Middle ground fruits and employed for flavoring and garnishing food, predominantly Eastern regions. Traditionally, sumac has been also used in popular medicine for the treatment of many ailments including hemorrhoids, wound healing, diarrhea, ulcers, and eye inflammation. Sumac drupes are indeed rich in various classes of phytochemicals including organic acids, flavonoids, tannins, and others, which are responsible of their powerful antioxidant capacity, from which treatment of many common diseases such as cardiovascular disease, diabetes, and cancer could benefit. In this work we evaluated the influence of fruit ripeness, conservation, and processing. To this aim, a phytochemical characterization of six different samples of *Rhus coriaria* L. was carried out. Specifically, headspace solid-phase micro extraction gas chromatography coupled to mass spectrometry and comprehensive two-dimensional liquid chromatography coupled to photodiode array and mass spectrometry detection, were employed. A total of 263 volatile compounds, including terpene hydrocarbons, acids, and aldehydes, as well as 83 polyphenolic compounds, mainly gallic acid derivatives, were positively identified. All samples showed a significant antioxidant activity by means of oxygen radical absorbance capacity, in line with their polyphenolic content and composition. Such findings set a solid ground to support the utilization of this plant as an attractive target for novel nutraceutical approaches and for drug discovery.

## 1. Introduction

*Rhus coriaria* L. (*R. coriaria*), commonly known as sumac, belongs to the Anacardiaceae family. According to “The Plant List” it is one of the 131 currently accepted species names of the very large and still under evaluation *Rhus* genus (The Plant List (2013). Version 1.1. published on the Internet http://www.theplantlist.org/) accessed on 7 January 2022 [1], to which are usually attributed more than 200 species by most authors [2,3,4].

Native to the Mediterranean and the Middle East regions, where it is a fairly common species, sumac has a wide distribution range in temperate and subtropical regions, extending from the Canary Islands, Azores, and Madeira in the west to Tadzhikistan and Afghanistan in the east [5]. Since ancient times, distinct parts of the plant have found several applications with significant technological value; tannins extracted from young stems, as well as from leaves, were utilized for tanning hides during leather preparation and in the past centuries the most extensive plantations have been indeed established for this purpose. Sumac has also counted as a source of natural dyes for the textile industry, yellow dye coming from young stems, brown from roots, black from leaves, and red from fruits. Especially bark and fruit preparations have been extensively used in popular medicine to obtain natural remedies against different affections such as eye and urinary tract infections, ulcer, diarrhea, and hepatic disorders [4,6,7]. Recently, *R. coriaria* has also gained some interest for its ornamental features that could be of value in urban landscaping and gardening [8]. Nonetheless, the most famous employment of sumac is to flavor and garnish food. In fact, dry drupes ground to powder are a typical spice that goes by the same plant common name: sumac. It is very popular in several Mediterranean and Middle East countries where it is used as a seasoning, flavoring, and acidulant ingredient in numerous traditional recipes and many significant biological activities have been ascribed to fruit-derived extracts, the most prominent being antioxidant, antimicrobial, and anticancer [7].

In terms of chemical characterization, as reported in the previous investigations, gas chromatography (GC) is the technique of choice for a reliable determination of volatile fingerprints and numerous are the sample preparation procedures that can be applied among these, headspace solid-phase microextraction (HS-SPME) coupled with GC has been widely accepted to the analysis of volatile components in various food matrices, due to its simplicity, selectivity, and sensitivity [9,10]. On the other hand, the polyphenolic content of natural products is usually assessed by liquid chromatography (LC) methods coupled to either a photodiode array (PDA) and/or mass spectrometry (MS) detection [11,12].

Sumac extracts have been characterized in terms of phytochemical composition: one of the earliest works was carried out in 1896 highlighting the presence of gallic acid and myricetin as a component of the leaf extract [13]. Afterwards, many other components were identified in different parts of the plant [7]; recently, more than 211 phytoconstituents including (iso)-flavonoids, tannins, terpenoids, anthocyanins, and others have been determined [14]. In this respect, complex mixtures of natural products are indeed very attractive for the potential of the synergistic beneficial effects of the components within the mixture, but they also pose a challenge concerning the variability of their composition in the starting plant material, due to factors such as geographical origin, environmental conditions, stages of fruit, and plant development and harvest. Therefore, the adoption of improved analytical tools is a mainstay for a precise characterization in which a solid proof of efficacy, together with the definition of a general mechanism of action, must stand.

In this work, the volatile profile and the polyphenolic content of six samples of sumac was carried out: samples one to four were obtained from fruits harvested in Sicily in different seasons and subjected to specific treatments, samples five and six were commercially available processed spices. Specifically, the volatile and non-volatile fraction of the fresh and dried Sicilian drupes and the commercial samples were compared in order to study, in detail, the influence of fruit ripeness, conservation, and processing on the phytochemical composition. As far as the analytical techniques are concerned, headspace solid-phase micro extraction (HS-SPME) gas chromatography (GC), and comprehensive two-dimensional liquid chromatography (LC×LC) were employed. Further, nutraceutical relevance of the characterized samples was assessed by oxygen radical absorbance capacity (ORAC); antioxidant activity is in fact one of the most prominent biological properties associated with sumac fruit and, in particular, with its primary derived food product, the sumac spice [7].

## 2. Results and Discussion

### 2.1. Volatile Fraction Analysis

Despite that, in the literature, there are several studies on *R. coriaria* fruits concerning their non-volatile metabolites, the volatile composition has been less investigated. Recently, Farag et al. reported the flavor profile results on sumac from Palestine, Jordan, and Egypt and its food products, analyzed via solid-phase microextraction (SPME) [15]. Brunke et al. identified over 120 constituents in the essential oil of *R. coriaria* fruits, among which terpene hydrocarbons, oxygenated terpenes, and aliphatic aldehydes were the most abundant [16]. The investigations of Giovannelli et al. on the aroma profile and essential oil composition of *R. coriaria* fruits from four Sicilian sites of collection allowed the identification of 106 compounds by SPME analysis and 169 in the essential oils by GC/MS, and the main constituents were revealed as being *p*-anisaldehyde, (*Z*)-2-heptenal, (*E*)-2-decenal, β-caryophllene, and cembrene [3].

**Figure 1:**
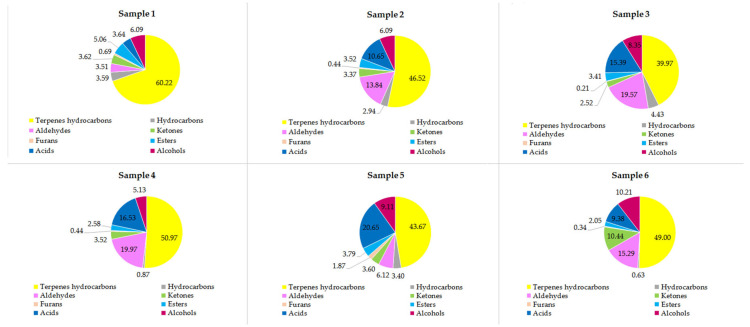
Percentage abundance of the compounds classes present in the analyzed samples. For samples description refer to Section 3.1.

The analysis carried out on the six samples led to the identification of 263 volatile compounds (Table S1), and to the best of our knowledge, this is the first time that the sumac flavor profile has been characterized so thoroughly. In particular, Sample 4 (99.47 ± 10.66) was the most abundant in terms of volatile compounds, whereas Sample 3 (86.71 ± 9.29) was the least abundant one. In terms of chemical classes, Sample 1 was the richest one in terpenes hydrocarbons, whereas it turned out to be the poorest in furans (Figure 1).

**Figure 2:**
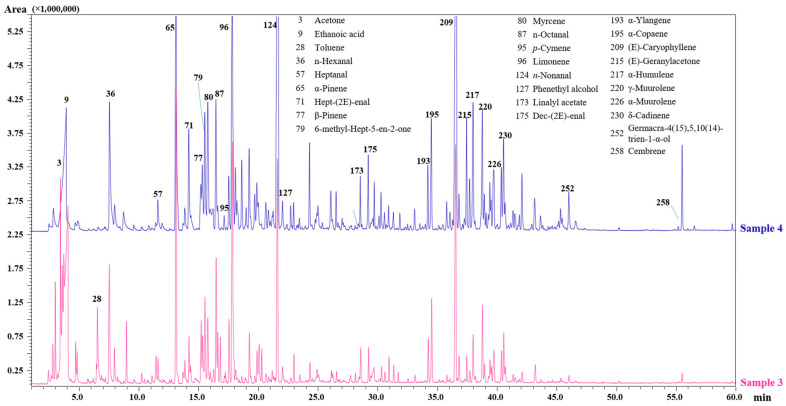
GC-MS analysis of the volatile profile for Sample 3 (fresh, collected in October) and Sample 4 (fresh and air-dried, collected in October).

Specifically in the fresh fruits, among the terpenes hydrocarbons, the most abundant compounds identified were α-pinene, (*E*)-caryophyllene, limonene, γ-muurolene, α-copaene, myrcene, *p*-cymene, β-pinene, δ-cadinene, methyl acetate, α-humulene, γ-terpinene, and α-phellandrene, followed by acids (ethanoic, formic, isovaleric) and aldehydes (*n*-nonanal, *n*-hexanal, *n*-octanal, (*E*)-2-heptenal). The sample harvested in the optimal ripening period featured a great relative area percentage for (*E*)-ß-Ocimene, (*Z*)-ß-Ocimene, *neo*-allo-ocimene, and (*Z*)-3-hexenyl-2-methylbutanoate. Instead, a very little amount of the same compounds has been encountered in the sample harvested in October. As shown in Figure 2, the fruits dried in the stove (Sample 4) exhibited a remarkable quantity of ethanoic acid, (*E*)-2-heptenal, myrcene, *n*-nonanal, α-copaene, (*E*)-caryophyllene, α-humulene and cembrene with respect to those not subjected to the drying process (Sample 3).

### 2.2. Polyphenols Analysis

**Figure 3:**
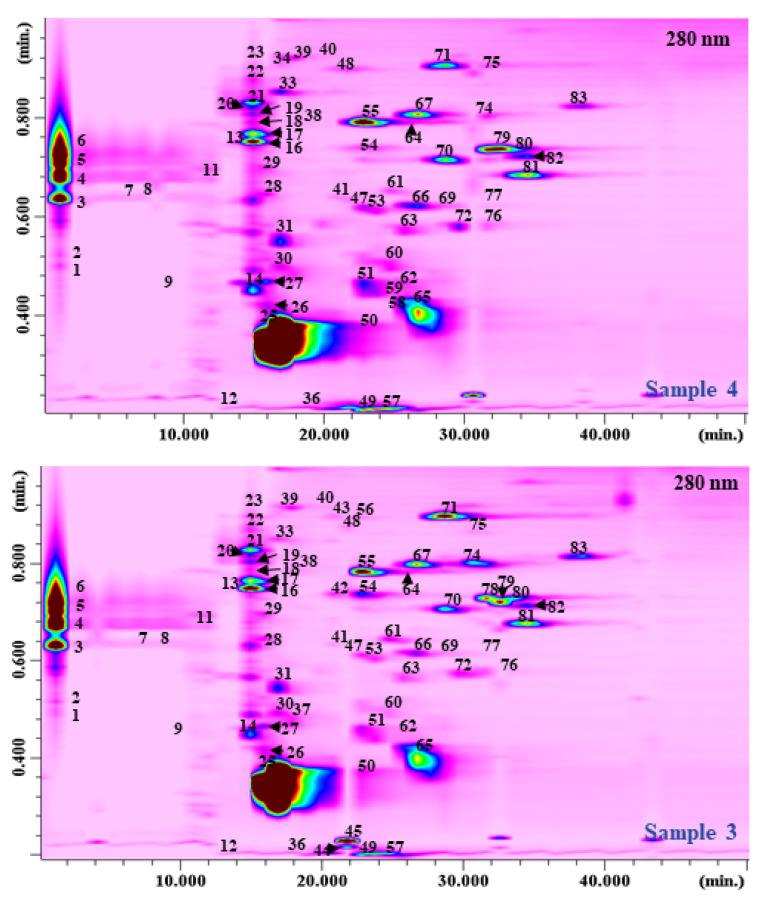
HILIC×RP-LC-PDA contour plots (280 nm) of the polyphenolic profile for Sample 3 (fresh, collected in October) and Sample 4 (fresh and air-dried, collected in October).

The polyphenolic fraction of *R. coriaria* fruits has been so far carried out by HPLC coupled with a photodiode array (PDA) and/or MS detection [17,18]. A comprehensive work on the phytochemical components of sumac fruit epicarp from Palestine by using HPLC-PDA-ESI-MS was reported by Abu-Reidah et al. [14], where a tentative identification of 211 phenolic and other phyto-constituents, most of which never reported before in *R. coriaria* fruits, were described. However, in none of these works was a quantification of the individual polyphenolic content reported due to the presence of overlapping peaks and matrix interferences. In this work the analysis of the polyphenolic compounds in *R. coriaria* samples was carried out by HILIC×RP-LC-PDA-ESI/MS. So far, most of the applications on polyphenols in food and natural products have been carried out on RP-LC×RP-LC [19,20,21,22,23,24,25,26,27], despite applications of HILIC×RP-LC also being reported [28,29,30]. Prior to either RP-LC×RP-LC or HILIC×RP-LC analysis, an optimization of the single separations must be carried out [31,32,33,34,35]. Normally a low mobile phase flow rate is used in the ^1^D separation to decrease the fraction volume onto the ^2^D and increase the ^1^D sampling rate; as a consequence, a microcolumn is used in the ^1^D. Since most commercial LC pumps are not capable of delivering a stable and repeatable flow rate, a higher flow rate is commonly employed and split up before entering the ^1^D column. In this work, an easy-to-use micropump with a completely new direct-drive engineering was employed and was capable of delivering stable micro- to semi-micro flow rates [25]. Notably when HILIC is hyphenated to RP, such coupling is not straightforward due to solvent incompatibility. To overcome such an issue, a modulation procedure called “active modulation” was reported [36,37]. Such an approach is based on the introduction of a make-up flow of a weaker solvent (water) after the ^1^D separation and before the entrance to the valves. In such a way, a reduction in the solvent strength is achieved, increasing the retention of the trap columns towards the compounds separated in the ^1^D. Afterwards, when the valve is actuated, the retained analytes are eluted in narrow bands thanks to the ^2^D mobile phase. Figure 3 reports the HILIC×RP-LC-PDA-ESI/MS plots of the polyphenolic fraction of *R. coriaria* for Samples 3 and 4. For MS detection, a triple quadruple MS analyzer was used, equipped with an electrospray interface working on both positive and negative ionization mode. The list of the compounds identified is reported in Table S2.

A total of 83 polyphenolic compounds were positively identified in the investigated samples by combining the information coming from PDA absorption (λ_max_), mass-to-charge ratio (*m*/*z*), and literature data [17,18]. Among them, the majority were represented by gallic acid and derivates (37), as well as quercetin derivates (11). The rest were represented by cyanidin, luteolin, myricetin, and apigenin derivates. Concerning the performance of the developed HILIC×RP-LC system, Table 1 reports the values attained for both peak capacity and orthogonality [38].

The highest theoretical peak capacity values, resulting from the product of the peak capacity, n_c_ of the two single dimensions [39], were attained for Sample 4 (3381), whereas the lowest one was attained for Sample 5 (2673). The orthogonality, A_O,_ values ranged from 0.72 to 0.90% for Sample 6 and Sample 3, respectively. With regards to corrected peak capacity ^2D^ n_corr_ values, incorporating under-sampling [40] and A_O_ values [38], the highest values were obtained for Samples 4 (1161) and 3 (1004), respectively. When comparing these values with previously reported ones, these were undoubtedly higher than RP-LC×RP-LC set-ups (695 in Wong et al. [22], 461–633 in Arena et al. [23], 404–639 in Arena et al. [26]), despite slightly lower than similar HILIC×RP-LC set-up with active flow modulation (1605–1830 in Toro-Uribe et al. [29]).

**Figure 4:**
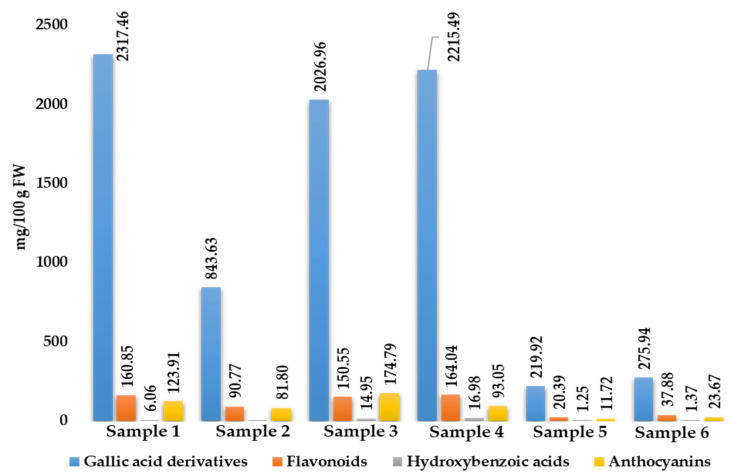
Quantitative content of the six *R. coriaria* samples investigated. For samples description refer to Section 3.1.

In terms of quantification, a semi-quantification approach was applied, taking into account the chemical classes of the identified compounds (Figure 4). Samples 1, 3, and 4 were the richest ones in terms of bioactive content, accounting for roughly 2608.28, 2367.25 and 2489.56 mg/100 g FW respectively; on the other hand, the poorest ones were represented by Sample 5 and 6, which were relative to commercial ones (253.28 and 338.86 mg/100 g FW, respectively). Notably, gallic acid derivatives were the most abundant ones in all samples investigated, ranging from 219.92 to 2317.46 mg/100 g FW.

### 2.3. Antioxidant Activity

**Figure 5:**
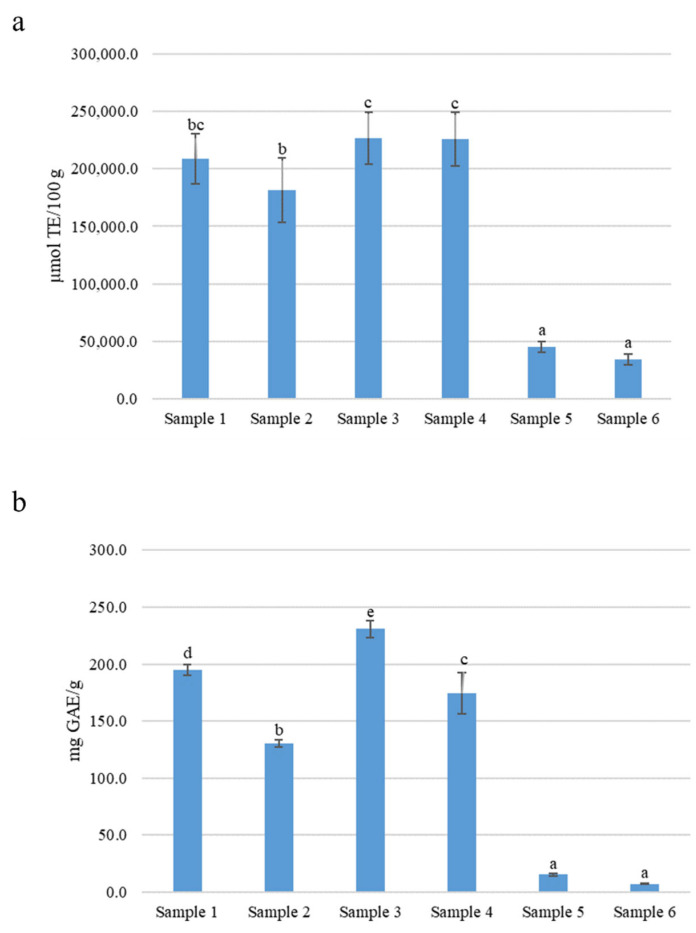
Antioxidant activity (**a**) and total phenolic content (**b**) of *R. coriaria* extracts. Antioxidant activity and total phenolic content were assessed by ORAC and Folin-Ciocalteau methods respectively; results are the average of three independent experiments and are expressed as µmol TE/100 g of extract (**a**) and as milligrams of gallic acid equivalents per gram of extract (mg GAE/g, (**b**). Letters labels indicate significant statistical differences among samples (*p* < 0.05) according to analysis of variance (ANOVA) and the Tukey’s (HSD) multiple range test. For samples description refer to Section 3.1.

Several studies reported a wide range of biological properties associated with *R. coriaria* fruit extracts such as antimicrobic, antiproliferative, antidiabetic, and, most prominently, antioxidant activity [7]. In this light, all samples were investigated by ORAC assay, one of the most common methods used to estimate the antioxidant capacity in food. Results, expressed as Trolox equivalents (µmol TE/100 g of extract), are presented in Figure 5a. Among extracts, Sample 3 (226,661.42 ± 22,867.89 µmol TE/100 g) and Sample 4 (225,836.14 ± 23,427.64 µmol TE/100 g) showed the highest antioxidant capacity, followed by Sample 1, Sample 2, Sample 5, and Sample 6 (respectively 208,709.88 ± 22,104.72, 181,393.61 ± 28,287.22, 44,978.42 ± 4717.30, and 34,321.05 ± 4456.08 µmol TE/100 g).

Furthermore, the total phenolic content (TPC) of *R. coriaria* extracts was determined by the Folin-Ciocalteau method. Results, expressed as milligrams of gallic acid equivalents (GAE) per gram of extract (mg GAE/g), are shown in Figure 5b. TPC of *R. coriaria* extracts well correlate with the antioxidant capacity. In general, the oven dried samples collected in Sicily, Sample 4, Sample 2, (respectively 174.24 ± 18.10, 130.28 ± 2.65 mg GAE/g), had a lower TCP when compared to their undried counterparts: Sample 3 (230.85 ± 7.37 mg GAE/g) and Sample 1 (194.72 ± 4.79 mg GAE/g). The lowest TCP was reported for the commercial samples, Sample 5 and Sample 6 (15.38 ± 1.40 and 7.25 ± 0.57 mg GAE/g, respectively).

## 3. Materials and Methods

### 3.1. Samples

A total of six sumac samples were analyzed. Samples 1 to 4 were collected in the territory of Licodia Eubea Municipality (37°09′ N, 14°42′ E), Sicily region (Italy), at an altitude of about 600 m above sea level from wild plants growing on soils belonging to the association ‘Regosols on sandy and conglomeratic rocks [41], the climate of this area, according to the Koppen and Geiger classification [42], is defined as ‘Csa, Hot-summer Mediterranean Climate’ with an average annual rainfall of 575 mm and an average annual temperature of 16.1 °C. Sample 1 consists of drupes harvested fresh in July, the most appropriate period as far as the ripening stage is concerned; Sample 2 were harvested at the same time but subsequently dried in a vacuum stove at the temperature of 40 °C. Sample 3 and 4 were collected in October (overripe stage), with the difference that also in this case Sample 4 was subjected to the same drying process previously reported.

Sample 5 and 6 were purchased as fruit dry powders on the internet (sumac spice), Sample 5 coming from the Mediterranean area without NaCl addition, and Sample 6 from Iran and with the addition of NaCl as a preservative.

### 3.2. Standard and Reagents

A C7-C40 Saturated Alkanes (1000 g/mL) standard mixture in hexane (49452-U) supplied by Merck Life Science (Merck KGaA, Darmstadt, Germany) was utilized for ALKANEs linear retention indices (LRIs) calculation.

Trolox (6-hydroxy-2,5,7,8-tetramethylchromane-2-carboxylic acid), gallic acid, AAPH (2,20-azobis(2-amidinopropane) dihydrochloride), fluorescein sodium salt, Folin-Ciocalteu reagent, sodium phosphate monobasic (NaH_2_PO_4_) and potassium phosphate dibasic (K_2_HPO_4_) were purchased from Merck Life Science (Merck KGaA, Darmstadt, Germany).

LC-MS-grade water, methanol, acetonitrile, and acetic acid were obtained from Merck Life Science (Merck KGaA, Darmstadt, Germany). Gallic acid, protocatechuic acid, isoquercetin, myricetin, and cyanidin were purchased from Merck Life Science (Merck KGaA, Darmstadt, Germany). Stock solutions of 1000 mg L^−1^ were prepared for each standard by dissolving 10 mg in 10 mL of methanol.

### 3.3. Sample Preparation

For the extraction method optimization, different sample weights, different solvents type and volumes, both pure and in mixture were tested for the polyphenol extraction. The highest yield was obtained, weighing 20 g of grinded sample (fresh or dried) in 100 mL of water as solvent and using an extraction temperature of 40 °C for 1 h.

In order to produce dry extract for HPLC analysis and examination antioxidant properties, liquid extracts were lyophilized. The aqueous samples were frozen at −80 °C for 1 h. Drying was carried out in a freeze dryer LyoQuest-55 (Telstar, Spain) at −50 °C and pressure of 0.011 mbar for 72 h. The yield of polyphenols was 13% *w*/*w*.

For both total phenolic content (TPC) and ORAC assays, lyophilized samples were dissolved in phosphate buffer (PBS, 75 mM, pH 7.0) (10 mg/mL) and filtered. Filtrate has been used straight away for analyses.

### 3.4. HS-SPME Extraction Conditions for the Detrmination of Volatiles

For the method optimization, a Carboxen/Polydimethylsiloxane (CAR/PDMS) 75 μm fiber 1 cm long (57343-U) and a divinylbenzene/carboxen/polydimethylsiloxane (DVB/CAR/PDMS) 50/30 μm fiber 1 cm long (57329-U), both purchased by Merck Life Science (Darmstadt, Germany), were tested. The fibers were conditioned before the initial use according to manufacturer’s instructions, and a cleaning step of 20 min at 10 °C below the fiber’s recommended maximum temperature was applied between consecutive analyses. Furthermore, sample conditioning times of 5 and 10 min were evaluated at the same temperature (37, 50, or 60 °C) employed for the extraction stage, and the analytical repeatability was excellent in both conditions. Different stirring rates (200 and 300 rpm) for sample conditioning and extraction have been also investigated. GC analyses were carried out using for each test using a 10 mL vial with 0.1, 0.2, and 0.5, g samples respectively, and the best results were obtained for a 0.2 g sample weight.

Three different fiber exposure times were also tested: 20, 30, and 40 min. The highest volatile extraction yield was achieved after an exposure time of 40 min, and most of the heavier molecular weight volatiles remained substantially stable thereafter.

In this investigation, the (DVB/CAR/PDMS) 50/30 μm fiber resulted in being the most useful in covering the wide range of volatile analytes; a conditioning time of 5 min and an extraction temperature of 60 °C resulted the best compromise between equilibration time and method sensitivity. Furthermore, a time of 40 min at the same temperature and stirring rate of 300 rpm have proven to be the best choice for an exhaustive extraction of the volatile components.

After the extraction, the analytes were thermally desorbed for 1 min at 260 °C in the GC injector port, in splitless mode.

### 3.5. GC–MS and GC-FID Analysis

The GC-MS and GC-FID analysis were carried out for qualitative and quantitative purposes, respectively.

GC-MS analyses were carried out on a GC-QP2010 system (Shimadzu, Kyoto, Japan). For the separation, an SLB-5ms fused-silica capillary column (30 m × 0.25 mm *i.d.* × 0.25 μm *df*) (29804-U) (Merck Life Science, Merck KGaA, Darmstadt, Germany) was applied. Helium was used as carrier gas at a constant linear velocity of 30.0 cm/s, which corresponded to an inlet pressure of 24.2 kPa. The temperature program was the following: 40 °C held for 1 min to 350 °C at 3 °C/min, held for 5 min. The interface and ion source temperatures were 250 and 200 °C, respectively. The acquisition was made in full scan mode in the mass range of 40–500 *m/z*, with a scanning rate interval of 0.2 s. Data handling was supported by GCMS solution ver. 4.30 software (Shimadzu, Kyoto, Japan). For the characterization, the following databases were used: W11N17 (Wiley11-Nist17, Wiley, Hoboken, NJ, USA, and FFNSC 4.0 (Shimadzu, Kyoto, Japan). The identification was performed applying two filters, namely spectral similarity match over 85% and linear retention index (LRI) match calculated using a C7–C40 saturated *n*-alkane homologue series with a filter window of ±10 LRI units.

GC-FID analyses were carried out on a GC2010 system (Shimadzu, Kyoto, Japan). Oven temperature program and injection parameters were the same as for MS applications. Helium was used as carrier gas, at a constant linear velocity of 30.0 cm/s, which corresponded to an inlet pressure of 97.4 kPa. The injector temperature was set at 260 °C. The FID temperature was set at 280 °C (sampling rate 200 ms), hydrogen and air flows were 40 and 400 mL/min, respectively. Data were collected by LabSolution software ver. 5.92 (Shimadzu, Kyoto, Japan). Quantitative results were determined as peak area percentage without any correction. Samples were analyzed in triplicates.

### 3.6. LC×LC-PDA/ESI-MS Analysis

LC×LC analyses were performed on a Shimadzu LC×LC instrument (Kyoto, Japan), consisting of a CBM-20A controller, one LC-Mikros binary pump, one LC-40BX3 dual-plunger parallel-flow pumps, one LC-30AD as make-up pump, a CTO-40C column oven, a SIL-40CX3 autosampler, and an SPD-M40 photo diode array (PDA) detector (1.0 μL detector flow cell volume). In order to connect the two dimensions, two high speed/high pressure two-position, six-ports switching valves with a micro-electric actuator (model FCV-32 AH, 1.034 bar; Shimadzu, Kyoto, Japan), equipped with two C18 guard columns, were employed. A third LC pump (LC-30AD) was connected through a t-piece between the outlet of the ^1^D and the inlet of switching valve. The LC×LC instrument was hyphenated to an LCMS-8050 mass spectrometer, through an ESI source (Shimadzu, Kyoto, Japan).

Separations were carried out on a ^1^D SEQuant ZIC-HILIC column (150 × 1.0 mm *I.D*., 3.5 µm *dp*) (Merck Life Science, Merck KGaA, Darmstadt, Germany) and a ^2^D Ascentis Express C18 column (50 × 4.6 mm *I.D*., 2.7 µm *dp*). (Merck Life Science, Merck KGaA, Darmstadt, Germany).

Two identical C18 guard columns (5 × 4.6 mm *I.D*., 5 µm *dp*) (Merck Life Science, Merck KGaA, Darmstadt, Germany); were used to collect and transfer the fractions from the ^1^D into the ^2^D.

^1^D mobile phases: (A) 0.1% formic acid in ACN, (B) 0.1% formic acid in water (pH 3). Gradient: 0 min, 30% B; 40 min, 60% B; 50 min, 100% B; 60 min, 100% B; 61 min, 30% B. Flow rate: 10 μL min^−1^. Column oven: 30 °C. Injection volume: 20 µL.

^2^D mobile phases: employed were (A) 0.1 % formic acid in water (pH 3), (B) 0.1% formic acid in ACN. Segmented-in-fraction conditions: (^1^D 0–12 min), 0.01 min, 10% B; 0.89 min, 40% B; 0.90 min, 10% B; (^1^D 13–17 min) 0.01 min, 0% B; 0.89 min, 40% B; 0.90 min, 0% B; (^1^D 18–51 min) 0.01 min, 0% B; 0.89 min, 25% B; 0.90 min, 0% B; Flow rate: 3 mL min^−1^. Modulation time: 1.00 min. Column oven: 30 °C. PDA conditions were in the range from 200 to 550 nm. Sampling rate was set to 40 Hz, whereas the time constant was acquired at 0.08 s.

ESI-MS conditions: mass spectral range: *m/z* 100–2000; event time: 1 s; nebulizing gas (N_2_) flow: 3 L min^−1^; drying gas (N_2_) flow: 10 L min^−1^; heating gas flow (air): 10 L min^−1^; heat block temperature: 400 °C; desolvation line (DL) temperature: 250 °C; interface temperature: 300 °C; interface voltage 3.50 kV; detector voltage: 1.80 kV.

The LC×LC-LCMS-8050 system and the switching valves were controlled by the Shimadzu Labsolution software (ver. 5.93). The LC×LC data were visualized and elaborated into two and three dimensions using Chromsquare ver. 2.3 software (Shimadzu, Kyoto, Japan).

Samples were diluted 1:4 with 0.1% formic acid in MeOH:ACN solution (70:30 *v/v*) prior to LC×LC-PDA/ESI-MS analysis.

For the quantitative analysis of polyphenolic compounds, gallic acid, protocatechuic acid, isoquercetin, myricetin, and cyanidin were employed. Standard calibration curves were prepared in a concentration range 10–500 mg L^−1^ with seven different concentration levels, run in triplicate.

### 3.7. Determination of Total Phenolic Content

TPC was determined by the Folin-Ciocalteau method, and the absorbance was measured using a microplate reader (Infinite^®^, 200 PRO multimode reader, Tecan, Männedorf, Switzerland) [43]. Extracts obtained were properly diluted and subsequently analyzed according to the literature [44]. Firstly, 20 µL of each extract, as well as standard (gallic acid) or blank (PBS), were mixed with 100 µL of Folin–Ciocalteau reagent in 1580 µL of PBS and incubated at room temperature for 8 min, in the dark. Then, 300 µL of Na_2_CO_3_ solution (0.2 g mL^−1^) were added and incubated at room temperature for 2 h, in the dark. Samples were centrifugated (20,817× *g* for 5 min at room temperature) and 200 µL of supernatants were transferred to a clear 96-well microplate; the absorbance was read at 765 nm.

The TPC was determined using a gallic acid standard curve (0−1000 µg/mL) (y = 0.0008x + 0.0031; R^2^ = 0.996). Analyses were performed in triplicate and results are expressed as milligrams of gallic acid equivalents (GAE) per gram of extract (mg GAE/g).

### 3.8. ORAC Assay

ORAC was determined as described by Zulueta et al. [45] using a multifunctional microplate reader (Infinite^®^, 200 PRO multimode reader, Tecan, Männedorf, Switzerland). The measurements were made in 96-well microplates with black sides and clear bottoms (BRANDplates^®^, Wertheim, Germany). Fluorescence was read with an excitation wavelength of 485 nm and an emission wavelength of 535 nm.

A stock solution of fluorescein (FL) was prepared dissolving 22 mg of FL in 50 mL of phosphate buffer (PBS, 75 mM, pH 7.0). FL solution was stored in complete darkness under refrigeration conditions. The FL working solution (7.7 µM) was prepared by diluting 0.167 mL of the stock solution in 25 mL of PBS. The AAPH solution (221 mM) was prepared by dissolving 600 mg of AAPH in 10 mL of PBS.

Samples obtained were properly diluted. In each well, 60 µL of FL working solution and 60 µL of sample, blank (PBS) or standard (Trolox) were mixed and incubated for 15 min at 37 °C. Then, 30 µL of AAPH solution was added. Fluorescence was measured immediately after the AAPH addition, and measurements were then taken every 5 min for 24 cycles at an incubation temperature of 37 °C.

The area under the curve (AUC), referred to by the fluorescence decay curve of each sample, blank, and Trolox, were calculated applying the following formula:

AUC = (0.5 + f5/f0 + f10/f0 + … + fn + 5/f0) × 5(1)

where f0 is the initial fluorescence, f5 is the fluorescence after 5 min, and fn is the fluorescence at time n. The net AUC was calculated by subtracting the AUC of the blank from the AUC of the sample. The antioxidant capacity of samples was determined using a Trolox calibration curve (6.25–100 µM) (y = 0.5926x; R^2^ = 0.996). Analyses were performed in triplicate and results are expressed as µmol TE/100 g of extract.

## 4. Conclusions

In this study, the phytochemical profile and antioxidant activity of six different fruit extracts of *R. coriaria* are reported. The volatile chemical profile was thoroughly investigated, revealing the presence of 263 volatile compounds; among them, the sample collected in October (overripe stage and dried in vacuum stove) was the most abundant in such compounds (99.47 ± 10.66), whereas the same sample, not subjected to the drying process, represented the least abundant one (86.71 ± 9.29). Moreover, a total of 83 polyphenolic compounds were positively identified in the investigated samples and among them, the majority were represented by gallic acid and its derivates (37). All samples showed an antioxidant activity consistent with polyphenolic content and composition. The obtained results highlight the importance of *R. coriaria* as a promising source of functional ingredients and boost its potential use in the food, nutraceutical, and pharmaceutical industries.

## Figures and Tables

**Table 1:** Peak capacity and orthogonality calculated for the HILIC×RP-LC focusing modulation set-up of the investigate samples.

	Sample 1	Sample 2	Sample 3	Sample 4	Sample 5	Sample 6
^1^D peak capacity, ^1^ n_c_	67	73	68	61	55	59
^2^D peak capacity, ^2^ n_c_	43	39	44	55	48	46
Theoretical peak capacity, ^2D^ n_c_	2875	2829	2971	3381	2673	2691
Effective peak capacity, ^2D^ n^I^_c_	1085	986	1114	1382	1181	1130
Orthogonality, A_O_	0.79	0.82	0.90	0.84	0.79	0.72
Corrected peak capacity, ^2D^ n_corr_	858	814	1004	1161	934	817

## Data Availability

The data presented in this study are available on request from the corresponding author.

## Supplementary Materials

The following are available online. Table S1. Most abundant volatile compounds contained in the samples analysed, expressed in area% as average of three measurements by GC-FID analysis, Table S2. Identification of the polyphenolic compounds in *R. coriaria* extracts by using HILIC×RP-LC-PDA/MS in positive and negative ionization mode.

## Sample Availability

Samples of the compounds are available from the authors.
