# Supplementary material for: Phytochemical Characterization of Rhus coriaria L. Extracts by Headspace Solid-Phase Micro Extraction Gas Chromatography, Comprehensive Two-Dimensional Liquid Chromatography, and Antioxidant Activity Evaluation
Source: Molecules. 2022 Mar 7;27(5):1727. doi: 10.3390/molecules27051727 (PMC8912007; doi:10.3390/molecules27051727)
Supplement: Supplementary file 1 [file molecules-27-01727-s001.zip › molecules-1622449-SI.pdf]

**Supplementary Materials.**

**Table S1.** Most abundant volatile compounds contained in the samples analysed, expressed in area% as average of three measurements by GC-FID analysis.

| | Compounds | $LRI_{ex}$ | $LRL_{rif}$ | Simil. % | Sample 1 | Sample 2 | Sample 3 | Sample 4 | Sample 5 | Sample 6 |
|---|---|---|---|---|---|---|---|---|---|---|
| 1 | Acetic aldehyde | 457 | - | 90 | 0.14 ± 0.02 | 0.03 ± 0.00 | 0.05 ± 0.01 | 0.06 ± 0.01 | 0.03 ± 0.00 | 0.02 ± 0.00 |
| 2 | Ethanol | 475 | - | 97 | 0.85 ± 0.09 | 0.36 ± 0.04 | 0.78 ± 0.08 | 0.14 ± 0.02 | 0.18 ± 0.02 | 0.20 ± 0.02 |
| 3 | Acetone | 491 | - | 97 | 0.32 ± 0.03 | 0.11 ± 0.01 | 0.21 ± 0.02 | 0.78 ± 0.08 | 0.10 ± 0.01 | 0.03 ± 0.00 |
| 4 | Formic acid | 529 | 480 | 90 | 0.67 ± 0.07 | 3.69 ± 0.40 | 3.18 ± 0.34 | 3.79 ± 0.41 | 0.23 ± 0.02 | 0.01 ± 0.00 |
| 5 | Methyl acetate | 566 | 633 | 90 | 0.06 ± 0.01 | 0.25 ± 0.03 | 0.98 ± 0.10 | - | 0.38 ± 0.04 | 0.01 ± 0.00 |
| 6 | 2,3-Butanedione | 581 | 561 | 90 | 0.11 ± 0.01 | 0.05 ± 0.01 | 0.10 ± 0.01 | - | 0.02 ± 0.00 | 0.01 ± 0.00 |
| 7 | 3-Methyl-2-pentanone | 578 | 745 | 90 | 0.06 ± 0.01 | 0.16 ± 0.02 | 0.04 ± 0.00 | - | 0.03 ± 0.00 | 0.04 ± 0.00 |
| 8 | 2-Methylfuran | 583 | 588 | 92 | 0.06 ± 0.01 | - | - | - | 1.21 ± 0.13 | 0.12 ± 0.01 |
| 9 | Ethanoic acid | 638 | 661 | 98 | 0.60 ± 0.06 | 10.47 ± 1.12 | 5.09 ± 0.55 | 11.16 ± 1.20 | 16.13 ± 1.73 | 8.19 ± 0.88 |
| 10 | Isobutyl alcohol | 735 | 621 | 94 | 0.01 ± 0.00 | 0.04 ± 0.00 | - | 0.03 ± 0.00 | 0.04 ± 0.00 | 0.12 ± 0.01 |
| 11 | (E)-2-Butenal | 641 | 650 | 91 | tr | 0.03 ± 0.00 | 0.06 ± 0.01 | 0.10 ± 0.01 | - | - |
| 12 | Butyl alcohol | 658 | 653 | 90 | tr | 0.11 ± 0.01 | 0.13 ± 0.01 | 0.03 ± 0.00 | - | - |
| 13 | 3-Hydroxy-pentene | 681 | 691 | 97 | 0.02 ± 0.00 | 0.01 ± 0.00 | 0.06 ± 0.01 | 0.10 ± 0.01 | - | - |
| 14 | Propyl methyl ketone | 685 | 682 | 90 | tr | 0.01 ± 0.00 | 0.58 ± 0.06 | 0.16 ± 0.02 | - | - |
| 15 | 3-Pentanone | 699 | 697 | 94 | 0.06 ± 0.01 | - | - | 0.10 ± 0.01 | - | - |
| 16 | *n*-Pentanal | 701 | 696 | 90 | 0.10 ± 0.01 | 0.08 ± 0.01 | 0.04 ± 0.00 | 0.07 ± 0.01 | - | 0.16 ± 0.02 |
| 17 | Pyruvic acid | 704 | 696 | 96 | 0.03 ± 0.00 | 0.05 ± 0.01 | 0.04 ± 0.00 | 0.06 ± 0.01 | 0.05 ± 0.01 | tr |
| 18 | Ethyl propanoate | 711 | 708 | 90 | 0.01 ± 0.00 | - | - | 0.02 ± 0.00 | - | tr |
| 19 | 3-Hydroxy-2-butanone | 716 | 716 | 91 | 0.35 ± 0.04 | - | - | 0.03 ± 0.00 | - | 0.03 ± 0.00 |
| 20 | Isoprenol | 730 | 724 | 93 | 0.09 ± 0.01 | 0.01 ± 0.00 | 0.01 ± 0.00 | 0.02 ± 0.00 | - | 0.01 ± 0.00 |
| 21 | Isopentyl alcohol | 734 | 729 | 97 | 0.06 ± 0.01 | - | 0.07 ± 0.01 | 0.05 ± 0.01 | 0.07 ± 0.01 | 0.07 ± 0.01 |
| 22 | *sec*-Butyl-carbinol | 737 | 733 | 92 | 0.03 ± 0.00 | - | 0.08 ± 0.01 | - | 0.10 ± 0.01 | - |
| 23 | Isopropyl ethyl ketone | 746 | 742 | 90 | 0.02 ± 0.00 | 0.01 ± 0.00 | 0.06 ± 0.01 | 0.03 ± 0.00 | - | - |
| 24 | 3-Hexanone | 746 | 782 | 90 | 0.03 ± 0.00 | - | 0.11 ± 0.01 | - | - | - |
| 25 | (Z)-2-Pentenal | 754 | - | 90 | tr | - | 0.03 ± 0.00 | - | - | - |
| 26 | (E)-2-Pentenal | 754 | 751 | 90 | 0.01 ± 0.00 | 0.02 ± 0.00 | 0.23 ± 0.02 | - | - | - |
| 27 | Isobutyric acid | 755 | 774 | 90 | - | - | - | 0.03 ± 0.00 | 0.05 ± 0.01 | - |
| 28 | Toluene | 764 | 763 | 94 | 1.04 ± 0.11 | 0.04 ± 0.00 | 1.99 ± 0.21 | 0.09 ± 0.01 | - | - |
| 29 | Pentyl alcohol | 767 | 763 | 95 | 0.03 ± 0.00 | 0.07 ± 0.01 | 0.16 ± 0.02 | - | 0.03 ± 0.00 | 0.16 ± 0.02 |
| 30 | (E)-2-Penten-1-ol | 769 | 761 | 90 | 0.02 ± 0.00 | - | 0.05 ± 0.01 | - | 0.02 ± 0.00 | - |
| 31 | Prenol | 776 | 772 | 90 | 0.01 ± 0.00 | - | 0.19 ± 0.02 | - | 0.09 ± 0.01 | - |
| 32 | Butanoic acid | 784 | 818 | 90 | 0.02 ± 0.00 | - | 0.11 ± 0.01 | 0.08 ± 0.01 | 0.06 ± 0.01 | 0.01 ± 0.00 |
| 33 | $\alpha$-Octene | 791 | 788 | 90 | 0.18 ± 0.02 | 0.12 ± 0.01 | 0.10 ± 0.01 | 0.04 ± 0.00 | 0.02 ± 0.00 | 0.03 ± 0.00 |
| 34 | 3-Methyl-crotonaldehyde | 789 | 780 | 90 | 0.01 ± 0.00 | 0.02 ± 0.00 | - | - | - | 0.01 ± 0.00 |
| 35 | *n*-Octane | 800 | 800 | 96 | 0.03 ± 0.00 | 0.01 ± 0.00 | 0.01 ± 0.00 | - | - | - |
| 36 | *n*-Hexanal | 802 | 801 | 96 | 0.07 ± 0.01 | 0.53 ± 0.06 | 2.88 ± 0.31 | 2.72 ± 0.29 | 0.10 ± 0.01 | 0.93 ± 0.10 |
| 37 | 1,3-Octadiene | 820 | 827 | 90 | 0.02 ± 0.00 | 0.12 ± 0.01 | - | 0.10 ± 0.01 | 0.01 ± 0.00 | 0.04 ± 0.00 |
| 38 | Furfural | 831 | 845 | 98 | 0.02 ± 0.00 | 0.43 ± 0.05 | - | - | 1.09 ± 0.12 | 0.16 ± 0.02 |
| 39 | Isovaleric acid | 835 | 842 | 90 | 0.14 ± 0.01 | 0.28 ± 0.03 | 0.88 ± 0.09 | - | - | 0.02 ± 0.00 |
| 40 | (E)-Ethyl crotonate | 843 | 839 | 93 | 0.01 ± 0.00 | 0.30 ± 0.03 | 0.02 ± 0.00 | 0.07 ± 0.01 | 0.09 ± 0.01 | - |
| 41 | Ethyl 2-methylbutyrate | 847 | 842 | 90 | 0.02 ± 0.00 | 0.08 ± 0.01 | 0.04 ± 0.00 | 0.03 ± 0.00 | 0.08 ± 0.01 | 0.01 ± 0.00 |
| 42 | (E)-2-Hexenal | 844 | 850 | 90 | - | 0.39 ± 0.04 | 0.01 ± 0.00 | 0.14 ± 0.02 | 0.07 ± 0.01 | - |
| 43 | (E)-3-Hexenol | 851 | 847 | 90 | 0.01 ± 0.00 | 0.14 ± 0.02 | 0.07 ± 0.01 | - | - | 0.15 ± 0.02 |
| 44 | (Z)-3-Hexenol | 854 | 853 | 96 | 0.52 ± 0.06 | 0.64 ± 0.07 | 0.04 ± 0.00 | - | 0.04 ± 0.00 | - |
| 45 | (E)-2-Hexenol | 866 | 864 | 90 | 0.01 ± 0.00 | 0.01 ± 0.00 | - | - | - | - |
| 46 | *n*-Hexanol | 869 | 867 | 96 | 0.12 ± 0.01 | 0.10 ± 0.01 | 0.17 ± 0.02 | 0.10 ± 0.01 | 0.26 ± 0.03 | 0.13 ± 0.01 |
| 47 | Isoamyl acetate | 875 | 871 | 97 | 0.03 ± 0.00 | 0.04 ± 0.00 | 0.03 ± 0.00 | 0.04 ± 0.00 | 0.30 ± 0.03 | - |
| 48 | 2-Methylbutyl acetate | 881 | 873 | 90 | 0.02 ± 0.00 | 0.04 ± 0.00 | 0.07 ± 0.01 | 0.01 ± 0.00 | 0.22 ± 0.02 | 0.15 ± 0.02 |
| 49 | *n*-Pentanoic acid | 882 | 911 | 90 | 0.02 ± 0.00 | - | - | 0.02 ± 0.00 | 0.01 ± 0.00 | 0.15 ± 0.02 |
| 50 | 3-Methyl-3-buten-1-yl-acetate | 884 | 878 | 90 | 0.02 ± 0.00 | 0.12 ± 0.01 | 0.03 ± 0.00 | 0.10 ± 0.01 | - | 0.08 ± 0.01 |
| 51 | 1-Buten-1-one | 885 | - | 90 | 0.02 ± 0.00 | - | 0.03 ± 0.00 | 0.17 ± 0.02 | - | - |
| 52 | Styrene | 886 | 891 | 90 | 0.01 ± 0.00 | - | 0.16 ± 0.02 | - | 0.33 ± 0.04 | - |
| 53 | Pentyl methyl ketone | 890 | 887 | 90 | 0.01 ± 0.00 | 0.14 ± 0.02 | - | - | - | - |
| 54 | Ethylbenzene | 859 | 857 | 90 | - | 0.05 ± 0.01 | 0.02 ± 0.00 | - | - | - |
| 55 | (E)-4-Heptenal | 894 | 901 | 90 | 0.02 ± 0.00 | 0.04 ± 0.00 | 0.04 ± 0.00 | - | - | - |
| 56 | *n*-Nonene | 895 | 890 | 90 | tr | 0.08 ± 0.01 | - | - | - | - |
| 57 | Heptanal | 903 | 906 | 94 | 0.04 ± 0.00 | 0.40 ± 0.04 | 0.52 ± 0.06 | 0.67 ± 0.07 | 0.14 ± 0.02 | 0.59 ± 0.06 |
| 58 | 2-Acetyl furan | 907 | 913 | 90 | 0.01 ± 0.00 | 0.12 ± 0.01 | - | 0.11 ± 0.01 | - | - |
| 59 | Tricyclene | 908 | 923 | 90 | 0.01 ± 0.00 | 0.06 ± 0.01 | 0.03 ± 0.00 | - | - | - |

| | | | | | | | | | |
|---|---|---|---|---|---|---|---|---|---|
| 60 | γ-Butyrolactone | 911 | 910 | 90 | 0.03 ± 0.00 | - | 0.10 ± 0.01 | - | - | 0.15 ± 0.02 |
| 61 | (E)-3-Nonene | 913 | 886 | 90 | 0.01 ± 0.00 | 0.06 ± 0.01 | - | - | 0.03 ± 0.00 | - |
| 62 | Pentyl acetate | 915 | 915 | 93 | 0.02 ± 0.00 | - | 0.01 ± 0.00 | - | 0.23 ± 0.02 | - |
| 63 | 3-Methyl-apopinene | 923 | 927 | 94 | 0.04 ± 0.00 | - | 0.06 ± 0.01 | 0.03 ± 0.00 | 0.01 ± 0.00 | 0.01 ± 0.00 |
| 64 | α-Thujene | 927 | 927 | 91 | 0.01 ± 0.00 | - | 0.08 ± 0.01 | 0.02 ± 0.00 | | 0.04 ± 0.00 |
| 65 | α-Pinene | 935 | 933 | 95 | 6.52 ± 0.70 | 3.64 ± 0.39 | 10.82 ± 1.16 | 6.94 ± 0.74 | 0.24 ± 0.03 | 1.61 ± 0.17 |
| 66 | Dihydromyrcene | 943 | 948 | 90 | 0.22 ± 0.02 | 0.01 ± 0.00 | 0.01 ± 0.00 | 0.02 ± 0.00 | 0.13 ± 0.01 | 0.08 ± 0.01 |
| 67 | α-Fenchene | 947 | 950 | 91 | 0.02 ± 0.00 | 0.02 ± 0.00 | 0.21 ± 0.02 | 0.14 ± 0.02 | - | 0.08 ± 0.01 |
| 68 | γ-Pentalactone | 949 | 954 | 92 | 0.04 ± 0.00 | 0.10 ± 0.01 | - | - | 0.27 ± 0.03 | 0.06 ± 0.01 |
| 69 | Camphene | 951 | 953 | 96 | 0.33 ± 0.04 | 0.36 ± 0.04 | 0.48 ± 0.05 | 0.43 ± 0.05 | - | 0.10 ± 0.01 |
| 70 | Thuja-2,4(10)-diene | 954 | 953 | 93 | 0.07 ± 0.01 | 0.06 ± 0.01 | 0.06 ± 0.01 | 0.05 ± 0.01 | - | 0.02 ± 0.00 |
| 71 | (E)-2-Heptenal | 958 | 956 | 94 | 0.14 ± 0.02 | 1.05 ± 0.11 | 0.92 ± 0.10 | 1.81 ± 0.19 | 0.25 ± 0.03 | 1.20 ± 0.13 |
| 72 | 5-Methyl furfural | 961 | 960 | 92 | 0.05 ± 0.01 | 0.03 ± 0.00 | 0.35 ± 0.04 | 0.06 ± 0.01 | - | - |
| 73 | Benzaldehyde | 964 | 960 | 90 | 0.08 ± 0.01 | 0.06 ± 0.01 | 0.03 ± 0.00 | 0.12 ± 0.01 | 0.87 ± 0.09 | 0.34 ± 0.04 |
| 74 | *n*-Heptanol | 972 | 970 | 90 | 0.05 ± 0.01 | 0.22 ± 0.02 | 0.06 ± 0.01 | 0.05 ± 0.01 | 0.09 ± 0.01 | 0.08 ± 0.01 |
| 75 | Sabinene | 973 | 972 | 91 | - | 0.09 ± 0.01 | 0.05 ± 0.01 | - | 0.04 ± 0.00 | 0.07 ± 0.01 |
| 76 | 1-Octen-3-one | 974 | 973 | 92 | - | 0.14 ± 0.02 | - | 0.34 ± 0.04 | 0.08 ± 0.01 | 0.11 ± 0.01 |
| 77 | β-Pinene | 978 | 978 | 97 | 1.54 ± 0.17 | - | 1.16 ± 0.12 | 0.05 ± 0.01 | 1.01 ± 0.11 | 0.04 ± 0.00 |
| 78 | Vinyl amyl carbinol | 982 | 978 | 90 | 0.24 ± 0.03 | 0.99 ± 0.11 | 1.09 ± 0.12 | - | - | 1.58 ± 0.17 |
| 79 | 6-Methyl-hept-5-en-2-one | 985 | 986 | 90 | 0.30 ± 0.03 | 1.28 ± 0.14 | 1.79 ± 0.19 | 1.30 ± 0.14 | 0.75 ± 0.08 | 1.07 ± 0.11 |
| 80 | Myrcene | 989 | 991 | 95 | 3.69 ± 0.40 | 2.28 ± 0.24 | 1.51 ± 0.16 | 2.14 ± 0.23 | 0.90 ± 0.10 | 1.72 ± 0.18 |
| 81 | 2-Pentyl-furan | 992 | 991 | 90 | 0.13 ± 0.01 | - | - | - | 0.24 ± 0.03 | - |
| 82 | 6-Methyl-hept-5-en-2-ol | 994 | 995 | 90 | 0.02 ± 0.00 | - | 0.14 ± 0.02 | 0.60 ± 0.06 | 0.24 ± 0.03 | - |
| 83 | *n*-Hexanoic acid | 995 | 997 | 90 | 0.03 ± 0.00 | 0.29 ± 0.03 | 0.06 ± 0.01 | 0.18 ± 0.02 | 0.09 ± 0.01 | 0.16 ± 0.02 |
| 84 | Mesitylene | 996 | 994 | 90 | - | - | - | 0.10 ± 0.01 | 0.10 ± 0.01 | 0.34 ± 0.04 |
| 85 | *n*-Decane | 1000 | 1000 | 93 | 0.07 ± 0.01 | 0.66 ± 0.07 | 0.20 ± 0.02 | - | 0.19 ± 0.02 | 0.27 ± 0.03 |
| 86 | 3-Octanol | 998 | 999 | 90 | - | - | - | - | 0.16 ± 0.02 | 0.91 ± 0.10 |
| 87 | *n*-Octanal | 1004 | 983 | 93 | 0.09 ± 0.01 | 3.37 ± 0.36 | 2.33 ± 0.25 | 2.08 ± 0.22 | 0.37 ± 0.04 | 1.86 ± 0.20 |
| 88 | (Z)-3-Hexenyl acetate | 1005 | 1008 | 97 | 0.66 ± 0.07 | - | - | - | - | 0.12 ± 0.01 |
| 89 | α-Phellandrene | 1007 | 1007 | 91 | 0.69 ± 0.07 | 0.44 ± 0.05 | 0.86 ± 0.09 | 0.43 ± 0.05 | 0.15 ± 0.02 | - |
| 90 | δ-3-Carene | 1010 | 1009 | 90 | 0.08 ± 0.01 | 0.23 ± 0.02 | - | - | 0.73 ± 0.08 | 0.69 ± 0.07 |
| 91 | Isopentyl isobutyrate | 1012 | 1014 | 92 | 0.16 ± 0.02 | 0.36 ± 0.04 | 0.05 ± 0.01 | - | 0.06 ± 0.01 | - |
| 92 | (E)-2-Hexenyl acetate | 1015 | 1017 | 93 | 0.05 ± 0.01 | 0.05 ± 0.01 | 0.77 ± 0.08 | 0.08 ± 0.01 | 0.45 ± 0.05 | 0.13 ± 0.01 |
| 93 | α-Terpinene | 1018 | 1018 | 94 | 0.45 ± 0.05 | 0.22 ± 0.02 | 0.11 ± 0.01 | 0.23 ± 0.02 | 0.21 ± 0.02 | 0.44 ± 0.05 |
| 94 | Pelargol | 1021 | 1200 | 90 | 0.18 ± 0.02 | 0.05 ± 0.01 | 0.17 ± 0.02 | 0.06 ± 0.01 | 0.12 ± 0.01 | 0.13 ± 0.01 |
| 95 | *p*-Cymene | 1025 | 1025 | 90 | 0.54 ± 0.06 | 0.74 ± 0.08 | 1.27 ± 0.14 | 0.87 ± 0.09 | 2.16 ± 0.23 | 2.97 ± 0.32 |
| 96 | Limonene | 1021 | 1030 | 97 | 4.71 ± 0.50 | 3.90 ± 0.42 | 6.24 ± 0.67 | 3.96 ± 0.42 | 3.28 ± 0.35 | 4.58 ± 0.49 |
| 97 | ß-Phellandrene | 1022 | 1031 | 96 | 1.14 ± 0.12 | 0.14 ± 0.01 | 0.19 ± 0.02 | 0.74 ± 0.08 | 0.42 ± 0.05 | 0.54 ± 0.06 |
| 98 | (Z)-ß-Ocimene | 1025 | 1035 | 90 | 2.72 ± 0.29 | 0.71 ± 0.08 | 0.08 ± 0.01 | 0.91 ± 0.10 | 0.41 ± 0.04 | 0.71 ± 0.08 |
| 99 | Oct-3-en-2-one | 1033 | 1036 | 90 | - | 0.09 ± 0.01 | 0.12 ± 0.01 | 0.15 ± 0.02 | 0.08 ± 0.01 | 0.53 ± 0.06 |
| 100 | 2(3H)-Furanone, dihydro-3-hydroxy-4,4-dimethyl-, | 1040 | 1032 | 92 | 0.35 ± 0.04 | 0.05 ± 0.01 | 0.05 ± 0.01 | 0.26 ± 0.03 | 0.35 ± 0.04 | 0.13 ± 0.01 |
| 101 | (Z)-2-Octenal | 1045 | 1047 | 90 | 0.08 ± 0.01 | 0.10 ± 0.01 | 0.02 ± 0.00 | 0.03 ± 0.00 | - | 0.22 ± 0.02 |
| 102 | Phenylacetaldehyde | 1046 | 1045 | 90 | - | 0.07 ± 0.01 | - | - | 0.18 ± 0.02 | - |
| 103 | (E)-ß-Ocimene | 1047 | 1046 | 96 | 5.48 ± 0.59 | 1.13 ± 0.12 | 0.10 ± 0.01 | 0.79 ± 0.08 | 0.19 ± 0.02 | 1.22 ± 0.13 |
| 104 | γ-Hexalactone | 1053 | 1060 | 90 | 0.02 ± 0.00 | 0.14 ± 0.02 | 0.01 ± 0.00 | 0.05 ± 0.01 | 0.16 ± 0.02 | 0.22 ± 0.02 |
| 105 | 4-Methyl decane | 1054 | 1060 | 90 | 0.03 ± 0.00 | 0.84 ± 0.09 | 0.13 ± 0.01 | 0.17 ± 0.02 | 0.45 ± 0.05 | 0.14 ± 0.02 |
| 106 | (E)-2-Octenal | 1056 | 1047 | 90 | 0.05 ± 0.01 | 1.68 ± 0.18 | 0.02 ± 0.00 | 1.11 ± 0.12 | 0.03 ± 0.00 | 0.28 ± 0.03 |
| 107 | γ-Terpinene | 1057 | 1058 | 94 | 0.67 ± 0.07 | 0.49 ± 0.05 | 0.95 ± 0.10 | 0.76 ± 0.08 | 0.52 ± 0.06 | 2.99 ± 0.32 |
| 108 | (E)-Decahydro-naphthalene | 1061 | 1062 | 93 | 0.08 ± 0.01 | 0.06 ± 0.01 | 0.13 ± 0.01 | 0.02 ± 0.00 | - | 1.20 ± 0.13 |
| 109 | 2-Methyl decane | 1062 | 1064 | 90 | 0.17 ± 0.02 | 0.32 ± 0.03 | 0.01 ± 0.00 | 0.14 ± 0.02 | 0.73 ± 0.08 | 0.13 ± 0.01 |
| 110 | (Z)-2-Octenol | 1068 | 1067 | 94 | - | 0.55 ± 0.06 | 0.16 ± 0.02 | 0.47 ± 0.05 | 0.19 ± 0.02 | 0.51 ± 0.05 |
| 111 | Octanol | 1072 | 1076 | 90 | 0.21 ± 0.02 | 1.82 ± 0.20 | 0.62 ± 0.07 | 0.93 ± 0.10 | - | 1.39 ± 0.15 |
| 112 | (Z)-Linalool oxide | 1075 | 1169 | 90 | 0.05 ± 0.01 | 1.10 ± 0.12 | 0.31 ± 0.03 | 0.52 ± 0.06 | 1.13 ± 0.12 | 0.12 ± 0.01 |
| 113 | Heptanoic acid | 1076 | 1116 | 92 | 0.06 ± 0.01 | 0.05 ± 0.00 | 0.03 ± 0.00 | 0.07 ± 0.01 | | 0.13 ± 0.01 |
| 114 | Terpinolene | 1080 | 1086 | 94 | 1.26 ± 0.14 | 1.30 ± 0.14 | 0.28 ± 0.03 | - | 0.08 ± 0.01 | 0.24 ± 0.03 |
| 115 | (E)-Linalool oxide | 1088 | 1086 | 90 | - | - | 0.04 ± 0.00 | 0.57 ± 0.06 | 0.60 ± 0.06 | 0.23 ± 0.02 |
| 116 | *p*-Cymenene | 1077 | 1093 | 90 | 0.80 ± 0.09 | 0.85 ± 0.09 | 0.19 ± 0.02 | 0.41 ± 0.04 | 0.50 ± 0.05 | 0.60 ± 0.06 |
| 117 | (E)-4-Nonenal | 1095 | 1098 | 90 | 0.02 ± 0.00 | 0.24 ± 0.03 | 0.08 ± 0.01 | 0.16 ± 0.02 | 0.05 ± 0.01 | 0.02 ± 0.00 |
| 118 | (3E,5E)-3,5-Octadien-2-one | 1096 | 1073 | 91 | 0.22 ± 0.02 | - | 0.03 ± 0.00 | 0.10 ± 0.01 | 0.20 ± 0.02 | 0.16 ± 0.02 |
| 119 | *n*-Undecane | 1100 | 1100 | 90 | - | 1.18 ± 0.13 | 0.25 ± 0.03 | 0.18 ± 0.02 | 0.98 ± 0.11 | 0.02 ± 0.00 |
| 120 | (Z)-3-Hexenyl propanoate | 1101 | 1101 | 92 | 0.07 ± 0.01 | - | 0.02 ± 0.00 | - | 0.07 ± 0.01 | - |
| 121 | 2-Methylbutyl-2-methyl-butyrate | 1102 | 1104 | 90 | 0.76 ± 0.08 | - | 0.01 ± 0.00 | - | - | 0.18 ± 0.02 |
| 122 | Linalool | 1103 | 1101 | 91 | - | 0.12 ± 0.01 | 0.16 ± 0.02 | - | 0.30 ± 0.03 | 0.67 ± 0.07 |

| | | | | | | | | | | |
|---|---|---|---|---|---|---|---|---|---|---|
| 123 | 3-Methylbutyl-2-methyl-butyrate | 1104 | 1104 | 92 | 0.04 ± 0.00 | - | 0.10 ± 0.01 | - | 0.12 ± 0.01 | - |
| 124 | *n*-Nonanal | 1105 | 1107 | 90 | 1.05 ± 0.11 | 5.69 ± 0.61 | 5.15 ± 0.55 | 7.84 ± 0.84 | 1.31 ± 0.14 | 5.14 ± 0.55 |
| 125 | 2-Methylbutyl isovalerate | 1108 | 1109 | 92 | 0.12 ± 0.01 | 0.13 ± 0.01 | - | 0.05 ± 0.01 | 0.06 ± 0.01 | 0.05 ± 0.01 |
| 126 | Heptyl acetate | 1112 | 1114 | 90 | 0.10 ± 0.01 | - | 0.04 ± 0.00 | 0.03 ± 0.00 | 0.10 ± 0.01 | 0.11 ± 0.01 |
| 127 | Phenethyl alcohol | 1115 | 1113 | 93 | 0.43 ± 0.05 | 0.51 ± 0.05 | 0.43 ± 0.05 | 0.49 ± 0.05 | 0.27 ± 0.03 | 0.10 ± 0.01 |
| 128 | *endo*-Fenchol | 1122 | 1119 | 90 | 0.12 ± 0.01 | 0.05 ± 0.01 | - | 0.23 ± 0.02 | 0.07 ± 0.01 | 0.06 ± 0.01 |
| 129 | *dihydro*-Citronellal | 1128 | 1125 | 90 | - | 0.01 ± 0.00 | 0.12 ± 0.01 | 0.04 ± 0.00 | 0.06 ± 0.01 | 0.03 ± 0.00 |
| 130 | Fenchyl alcohol | 1123 | 1123 | 90 | 0.14 ± 0.02 | 0.18 ± 0.02 | 0.04 ± 0.00 | 0.03 ± 0.00 | 0.05 ± 0.01 | 0.03 ± 0.00 |
| 131 | *allo*-(4E,6Z)-Ocimene | 1129 | 1128 | 90 | 0.33 ± 0.04 | 0.23 ± 0.02 | 0.01 ± 0.00 | 0.37 ± 0.04 | 0.25 ± 0.03 | 0.27 ± 0.03 |
| 132 | 2-Vinyl anisole | 1132 | 1135 | 91 | - | 0.06 ± 0.01 | - | 0.07 ± 0.01 | - | - |
| 133 | Cosmene | 1133 | 1131 | 90 | 0.69 ± 0.07 | 0.03 ± 0.00 | 0.01 ± 0.00 | - | - | 0.04 ± 0.00 |
| 134 | 3-Nonen-2-one | 1140 | 1137 | 90 | 0.70 ± 0.08 | 0.14 ± 0.02 | 0.02 ± 0.00 | 0.08 ± 0.01 | 0.09 ± 0.01 | 0.02 ± 0.00 |
| 135 | *neo-allo*-Ocimene | 1141 | 1145 | 90 | 1.33 ± 0.14 | 0.26 ± 0.03 | 0.01 ± 0.00 | 0.10 ± 0.01 | 0.07 ± 0.01 | 0.04 ± 0.00 |
| 136 | (E)-Pinocarveol | 1142 | 1141 | 94 | 0.23 ± 0.02 | 0.01 ± 0.00 | 0.19 ± 0.02 | 0.23 ± 0.02 | 0.29 ± 0.03 | 0.09 ± 0.01 |
| 137 | (Z)-2-Nonenal | 1148 | 1148 | 90 | 0.35 ± 0.04 | 0.07 ± 0.01 | 0.02 ± 0.00 | 0.08 ± 0.01 | 0.08 ± 0.01 | 0.06 ± 0.01 |
| 138 | (Z)-β-Terpineol | 1149 | 1149 | 91 | 0.21 ± 0.02 | 0.12 ± 0.01 | 0.01 ± 0.00 | 0.01 ± 0.00 | 0.05 ± 0.01 | 0.12 ± 0.01 |
| 139 | 6-Methyl undecane | 1151 | 1152 | 90 | 0.20 ± 0.02 | - | 0.01 ± 0.00 | 0.11 ± 0.01 | 0.14 ± 0.02 | 0.07 ± 0.01 |
| 140 | Neomenthol | 1155 | 1170 | 92 | 0.38 ± 0.04 | 0.17 ± 0.02 | - | 0.03 ± 0.00 | 0.10 ± 0.01 | 0.36 ± 0.04 |
| 141 | Camphene hydrate | 1156 | 1156 | 90 | 0.70 ± 0.08 | 0.03 ± 0.00 | 0.03 ± 0.00 | 0.02 ± 0.00 | tr | - |
| 142 | (E)-2-Nonenal | 1162 | 1163 | 96 | 0.11 ± 0.01 | 0.81 ± 0.09 | 0.37 ± 0.04 | 1.12 ± 0.12 | 0.09 ± 0.01 | 0.68 ± 0.07 |
| 143 | Pinocarvone | 1166 | 1164 | 90 | 0.56 ± 0.06 | 0.12 ± 0.01 | 0.08 ± 0.01 | 0.13 ± 0.01 | 0.11 ± 0.01 | 0.25 ± 0.03 |
| 144 | 3-Methyl undecane | 1167 | 1170 | 91 | 0.75 ± 0.08 | - | 0.02 ± 0.00 | 0.01 ± 0.00 | - | 0.08 ± 0.01 |
| 145 | Isoborneol | 1168 | 1165 | 94 | 0.05 ± 0.01 | 0.14 ± 0.02 | 0.11 ± 0.01 | 0.19 ± 0.02 | 0.34 ± 0.04 | 0.13 ± 0.01 |
| 146 | Nonanol | 1174 | 1176 | 90 | 0.08 ± 0.01 | 0.40 ± 0.04 | 0.30 ± 0.03 | 0.29 ± 0.03 | 0.57 ± 0.06 | 0.04 ± 0.00 |
| 147 | Borneol | 1176 | 1173 | 91 | 0.47 ± 0.05 | 0.88 ± 0.09 | 0.19 ± 0.02 | 0.50 ± 0.05 | 0.58 ± 0.06 | 0.12 ± 0.01 |
| 148 | (Z)-Pinocamphone | 1180 | 1176 | 92 | - | - | 0.03 ± 0.00 | 0.09 ± 0.01 | 0.11 ± 0.01 | 0.06 ± 0.01 |
| 149 | Octanoic acid | 1181 | 1192 | 91 | 0.43 ± 0.05 | 0.11 ± 0.01 | - | - | - | 0.02 ± 0.00 |
| 150 | 4-Terpinenol | 1183 | 1184 | 90 | - | 0.17 ± 0.02 | 0.04 ± 0.00 | 0.14 ± 0.02 | 0.69 ± 0.07 | 0.52 ± 0.06 |
| 151 | (Z)-3-Hexenyl-butyrate | 1185 | 1187 | 91 | 1.94 ± 0.21 | 0.05 ± 0.01 | - | 0.06 ± 0.01 | 0.17 ± 0.02 | 0.03 ± 0.00 |
| 152 | 1-Dodecene | 1190 | 1191 | 90 | 0.74 ± 0.08 | - | - | - | 0.18 ± 0.02 | 0.01 ± 0.00 |
| 153 | Octyl methyl ketone | 1194 | 1196 | 91 | 0.86 ± 0.09 | 0.26 ± 0.03 | 0.01 ± 0.00 | 0.06 ± 0.01 | 0.05 ± 0.01 | 0.12 ± 0.01 |
| 154 | (E)-4-Decenal | 1195 | 1197 | 92 | 0.29 ± 0.03 | 0.11 ± 0.01 | 0.03 ± 0.00 | 0.02 ± 0.00 | 0.11 ± 0.01 | 0.08 ± 0.01 |
| 155 | α-Terpineol | 1198 | 1195 | 95 | 1.07 ± 0.11 | 1.85 ± 0.20 | 0.24 ± 0.03 | 0.57 ± 0.06 | 1.18 ± 0.13 | 1.17 ± 0.13 |
| 156 | *n*-Dodecane | 1200 | 1200 | 92 | 1.02 ± 0.11 | 0.78 ± 0.08 | 0.19 ± 0.02 | 0.21 ± 0.02 | - | - |
| 157 | 1,6-Dihydrocarveol | 1201 | 1203 | 91 | 0.86 ± 0.09 | 0.03 ± 0.00 | - | - | 3.28 ± 0.35 | 1.30 ± 0.14 |
| 158 | (E)-Dihydrocarvone | 1204 | 1204 | 90 | 0.24 ± 0.03 | - | - | - | 0.58 ± 0.06 | 0.15 ± 0.02 |
| 159 | Decanal | 1207 | 1208 | 93 | 0.07 ± 0.01 | 0.52 ± 0.06 | 0.18 ± 0.02 | 0.51 ± 0.05 | - | 0.87 ± 0.09 |
| 160 | Verbenone | 1208 | 1205 | 90 | 0.38 ± 0.04 | 0.05 ± 0.01 | - | - | - | 0.08 ± 0.01 |
| 161 | Octyl acetate | 1210 | 1214 | 90 | 0.01 ± 0.00 | 0.09 ± 0.01 | 0.05 ± 0.01 | 0.14 ± 0.02 | 0.36 ± 0.04 | 0.10 ± 0.01 |
| 162 | 2,5-Dimethyl-undecane | 1211 | 1210 | 90 | 0.21 ± 0.02 | 0.08 ± 0.01 | 0.05 ± 0.01 | 0.05 ± 0.01 | 0.18 ± 0.02 | 0.02 ± 0.00 |
| 163 | (2E,4E)-Nonadienal | 1218 | 1218 | 91 | 0.04 ± 0.00 | 0.13 ± 0.01 | 0.02 ± 0.00 | 0.18 ± 0.02 | 0.09 ± 0.01 | 0.40 ± 0.04 |
| 164 | Fenchyl acetate | 1220 | 1219 | 90 | 0.01 ± 0.00 | 0.08 ± 0.01 | 0.05 ± 0.01 | 0.11 ± 0.01 | - | 0.09 ± 0.01 |
| 165 | Citronellyl nitrile | 1221 | 1221 | 90 | 0.13 ± 0.01 | 0.24 ± 0.03 | - | 0.11 ± 0.01 | 0.08 ± 0.01 | 0.25 ± 0.03 |
| 166 | (E)-*p*-Mentha-1(7),8-dien-2-ol | 1226 | 1230 | 91 | 0.09 ± 0.01 | 0.07 ± 0.01 | - | - | 0.14 ± 0.02 | 0.08 ± 0.01 |
| 167 | Decane, 5-ethyl-5-methyl- | 1229 | - | 92 | 0.13 ± 0.01 | 0.08 ± 0.01 | 0.04 ± 0.00 | 0.01 ± 0.00 | 0.16 ± 0.02 | 0.22 ± 0.02 |
| 168 | (Z)-3-Hexenyl-2-methylbutanoate | 1230 | 1231 | 90 | 1.06 ± 0.11 | 0.32 ± 0.03 | 0.08 ± 0.01 | 0.05 ± 0.01 | 0.06 ± 0.01 | 0.03 ± 0.00 |
| 169 | 6-Ethyl-undecane | 1231 | 1230 | 93 | 0.47 ± 0.05 | 0.24 ± 0.03 | 0.16 ± 0.02 | 0.07 ± 0.01 | 0.10 ± 0.01 | 0.15 ± 0.02 |
| 170 | Cuminaldehyde | 1240 | 1243 | 90 | 0.57 ± 0.06 | 0.03 ± 0.00 | 0.01 ± 0.00 | 0.14 ± 0.02 | 0.21 ± 0.02 | 0.27 ± 0.03 |
| 171 | Carvone | 1245 | 1246 | 90 | 0.09 ± 0.01 | 0.05 ± 0.01 | 0.04 ± 0.00 | 0.01 ± 0.00 | 1.61 ± 0.17 | 7.55 ± 0.81 |
| 172 | (Z)-2-Decenal | 1248 | 1250 | 91 | 0.09 ± 0.01 | 0.17 ± 0.02 | 0.03 ± 0.00 | 0.04 ± 0.00 | 0.14 ± 0.02 | 0.06 ± 0.01 |
| 173 | Linalyl acetate | 1250 | 1250 | 92 | 0.86 ± 0.09 | 0.15 ± 0.02 | 0.65 ± 0.07 | 0.68 ± 0.07 | 0.19 ± 0.02 | 0.01 ± 0.00 |
| 174 | Ionane | 1253 | 1246 | 92 | 0.47 ± 0.05 | 0.14 ± 0.02 | 0.02 ± 0.00 | 0.10 ± 0.01 | 0.08 ± 0.01 | 0.06 ± 0.01 |
| 175 | (E)-2-Decenal | 1264 | 1265 | 94 | 0.56 ± 0.06 | 2.94 ± 0.32 | 0.59 ± 0.06 | 0.95 ± 0.10 | 0.19 ± 0.02 | 1.55 ± 0.17 |
| 176 | Nonanoic acid | 1274 | 1289 | 95 | 0.08 ± 0.01 | 0.04 ± 0.00 | 0.38 ± 0.04 | 0.27 ± 0.03 | 0.30 ± 0.03 | 0.03 ± 0.00 |
| 177 | Bornyl acetate | 1287 | 1285 | 96 | 0.97 ± 0.10 | 0.29 ± 0.03 | 0.32 ± 0.03 | 0.46 ± 0.05 | 0.29 ± 0.03 | 0.28 ± 0.03 |
| 178 | 2-*tert*-Butyl-,(Z)-cyclohexanol acetate | 1295 | 1291 | 90 | 0.04 ± 0.00 | 0.15 ± 0.02 | 0.20 ± 0.02 | 0.27 ± 0.03 | 0.03 ± 0.00 | 0.71 ± 0.08 |
| 179 | 1-Tridecene | 1297 | 1292 | 90 | 0.36 ± 0.04 | 0.18 ± 0.02 | - | 0.08 ± 0.01 | 0.41 ± 0.04 | 0.01 ± 0.00 |
| 180 | *n*-Tridecane | 1300 | 1300 | 91 | 0.07 ± 0.01 | 0.36 ± 0.04 | 0.02 ± 0.00 | 0.02 ± 0.00 | 0.83 ± 0.09 | 0.03 ± 0.00 |
| 181 | 1-Terpinen-4-yl acetate | 1301 | 1296 | 90 | 0.13 ± 0.01 | 0.52 ± 0.06 | 0.01 ± 0.00 | 0.27 ± 0.03 | - | 0.01 ± 0.00 |
| 182 | Carvacrol | 1302 | 1300 | 91 | 0.08 ± 0.01 | 0.06 ± 0.01 | 0.38 ± 0.04 | 0.02 ± 0.00 | 0.30 ± 0.03 | 0.80 ± 0.09 |
| 183 | *n*-Undecanal | 1309 | 1309 | 90 | 0.01 ± 0.00 | 0.42 ± 0.05 | 0.02 ± 0.00 | 0.03 ± 0.00 | 0.56 ± 0.06 | 0.46 ± 0.05 |
| 184 | Dihydro citronellol acetate | 1312 | 1319 | 91 | 0.09 ± 0.01 | 0.21 ± 0.02 | 0.17 ± 0.02 | 0.08 ± 0.01 | 0.29 ± 0.03 | 0.07 ± 0.01 |
| 185 | (Z)-3-Hexenyl tiglate | 1322 | 1325 | 93 | 0.53 ± 0.06 | 1.04 ± 0.11 | 0.02 ± 0.00 | 0.04 ± 0.00 | 0.22 ± 0.02 | 0.33 ± 0.04 |

| | | | | | | | | | | |
|---|---|---|---|---|---|---|---|---|---|---|
| 186 | γ-Terpinyl acetate | 1349 | 1358 | 91 | 0.16 ± 0.02 | 0.26 ± 0.03 | 0.14 ± 0.02 | 0.28 ± 0.03 | 0.55 ± 0.06 | 0.25 ± 0.03 |
| 187 | 5-Methyl tridecane | 1350 | 1348 | 92 | 0.07 ± 0.01 | 0.04 ± 0.00 | 0.02 ± 0.00 | - | 0.04 ± 0.00 | 0.03 ± 0.00 |
| 188 | Eugenol | 1351 | 1357 | 90 | 0.01 ± 0.00 | - | 0.03 ± 0.00 | 0.01 ± 0.00 | 0.23 ± 0.02 | 0.01 ± 0.00 |
| 189 | (Z)-Geranyl acetate | 1355 | 1361 | 91 | 0.02 ± 0.00 | 0.03 ± 0.00 | 0.05 ± 0.01 | 0.08 ± 0.01 | - | - |
| 190 | 2(3H)-Furanone, dihydro-5-pentyl- | 1359 | 1362 | 90 | 0.09 ± 0.01 | 0.01 ± 0.00 | 0.04 ± 0.00 | 0.01 ± 0.00 | 0.08 ± 0.01 | 0.09 ± 0.01 |
| 191 | (Z)-8-Undecenal | 1361 | 1365 | 93 | 0.05 ± 0.01 | 0.13 ± 0.01 | 0.04 ± 0.00 | 0.03 ± 0.00 | 0.19 ± 0.02 | 0.14 ± 0.02 |
| 192 | Isoledene | 1369 | 1372 | 91 | 0.25 ± 0.03 | 0.04 ± 0.00 | 0.01 ± 0.00 | 0.10 ± 0.01 | 0.19 ± 0.02 | 0.08 ± 0.01 |
| 193 | α-Ylangene | 1373 | 1371 | 90 | 0.51 ± 0.05 | 0.53 ± 0.06 | 0.79 ± 0.08 | 0.91 ± 0.10 | 0.24 ± 0.03 | 0.52 ± 0.06 |
| 194 | Cyclosativene | 1377 | 1367 | 90 | 0.33 ± 0.04 | - | 0.02 ± 0.00 | - | 0.62 ± 0.07 | 0.04 ± 0.00 |
| 195 | α-Copaene | 1380 | 1375 | 92 | 1.00 ± 0.11 | 0.98 ± 0.11 | 1.56 ± 0.17 | 1.82 ± 0.20 | 0.06 ± 0.01 | 0.69 ± 0.07 |
| 196 | β-Bourbonene | 1381 | 1382 | 90 | 0.13 ± 0.01 | 0.05 ± 0.01 | 0.01 ± 0.00 | 0.02 ± 0.00 | 0.25 ± 0.03 | 0.32 ± 0.03 |
| 197 | β-Elemene | 1387 | 1390 | 90 | 0.16 ± 0.02 | 0.20 ± 0.02 | 0.02 ± 0.00 | - | - | 0.13 ± 0.01 |
| 198 | α-Gurjunene | 1395 | 1406 | 92 | - | - | 0.04 ± 0.00 | 0.07 ± 0.01 | 0.02 ± 0.00 | 0.01 ± 0.00 |
| 199 | Sativene | 1397 | 1394 | 93 | 0.13 ± 0.01 | 0.05 ± 0.01 | 0.02 ± 0.00 | 0.03 ± 0.00 | 0.03 ± 0.00 | 0.04 ± 0.00 |
| 200 | *n*-Tetradecane | 1400 | 1400 | 91 | 0.17 ± 0.02 | 0.71 ± 0.08 | 0.04 ± 0.00 | - | 0.18 ± 0.02 | 0.06 ± 0.01 |
| 201 | Decyl methyl ketone | 1401 | 1393 | 90 | 0.18 ± 0.02 | - | 0.04 ± 0.00 | 0.03 ± 0.00 | 0.05 ± 0.01 | 0.02 ± 0.00 |
| 202 | Dodecanal | 1404 | 1410 | 91 | - | - | - | 0.02 ± 0.00 | - | 0.01 ± 0.00 |
| 203 | (Z)-Caryophyllene | 1410 | 1413 | 90 | 0.47 ± 0.05 | 0.11 ± 0.01 | - | 0.47 ± 0.05 | 0.47 ± 0.05 | 0.04 ± 0.00 |
| 204 | β-Longipinene | 1416 | 1405 | 92 | 0.14 ± 0.02 | - | 0.08 ± 0.01 | 0.34 ± 0.04 | 0.03 ± 0.00 | - |
| 205 | Bicyclo [5.2.0]nonane, 2-methylene-4,8,8-trimethyl-4-vinyl- | 1418 | - | 90 | 0.04 ± 0.00 | 1.01 ± 0.11 | 0.15 ± 0.02 | 0.16 ± 0.02 | 0.11 ± 0.01 | 0.43 ± 0.05 |
| 206 | Bicyclo[5.2.0]nonane, 4-methylene-2,8,8-trimethyl-2-vinyl- | 1419 | - | 90 | 0.47 ± 0.05 | 0.24 ± 0.03 | 0.02 ± 0.00 | - | 0.06 ± 0.01 | 0.26 ± 0.03 |
| 207 | Isocaryophyllene | 1420 | 1419 | 91 | 0.23 ± 0.02 | - | - | - | 0.13 ± 0.01 | 0.05 ± 0.01 |
| 208 | γ-Elemene | 1421 | 1432 | 90 | 0.05 ± 0.01 | 0.33 ± 0.04 | 0.01 ± 0.00 | - | - | 0.07 ± 0.01 |
| 209 | (E)-Caryophyllene | 1425 | 1425 | 96 | 4.78 ± 0.51 | 5.73 ± 0.61 | 8.31 ± 0.89 | 9.28 ± 0.99 | 11.62 ± 1.24 | 12.47 ± 1.34 |
| 210 | α-Guaiene | 1435 | 1438 | 93 | 4.12 ± 0.44 | 0.01 ± 0.00 | 0.02 ± 0.00 | - | tr | - |
| 211 | 10,10-Dimethyl-2,6-dimethylenebicyclo[7.2.0]undecane | 1433 | 1440 | 91 | - | 0.02 ± 0.00 | 0.40 ± 0.04 | 0.58 ± 0.06 | 0.43 ± 0.05 | 0.75 ± 0.08 |
| 212 | Isopentyl benzoate | 1442 | 1439 | 93 | 0.38 ± 0.04 | 0.11 ± 0.01 | 0.01 ± 0.00 | 0.11 ± 0.01 | 0.18 ± 0.02 | 0.05 ± 0.01 |
| 213 | (E)-α-Bergamotene | 1434 | 1432 | 90 | - | 0.03 ± 0.00 | 0.03 ± 0.00 | - | tr | 0.01 ± 0.00 |
| 214 | Aromadendrene | 1451 | 1438 | 90 | 0.04 ± 0.00 | 0.04 ± 0.00 | 0.04 ± 0.00 | 0.04 ± 0.00 | 0.03 ± 0.00 | - |
| 215 | (E)-Geranylacetone | 1453 | 1450 | 95 | 0.29 ± 0.03 | 1.34 ± 0.14 | 0.41 ± 0.04 | 1.59 ± 0.17 | 0.66 ± 0.07 | 0.45 ± 0.05 |
| 216 | (E)-9-epi-Caryophyllene | 1456 | 1464 | 90 | 0.37 ± 0.04 | 0.45 ± 0.05 | 0.24 ± 0.03 | 0.77 ± 0.08 | 0.74 ± 0.08 | 0.69 ± 0.07 |
| 217 | α-Humulene | 1463 | 1454 | 95 | 1.48 ± 0.16 | 1.05 ± 0.11 | 0.95 ± 0.10 | 2.19 ± 0.23 | 2.52 ± 0.27 | 2.32 ± 0.25 |
| 218 | Guaia-6,9-diene | 1452 | - | 91 | - | 0.01 ± 0.00 | 0.02 ± 0.00 | 0.03 ± 0.00 | 0.02 ± 0.00 | - |
| 219 | Germacrene D | 1479 | 1480 | 92 | 0.05 ± 0.01 | 0.12 ± 0.01 | 0.12 ± 0.01 | 0.14 ± 0.02 | 0.20 ± 0.02 | 0.08 ± 0.01 |
| 220 | γ-Muurolene | 1481 | 1478 | 94 | 0.69 ± 0.07 | 1.11 ± 0.12 | 1.57 ± 0.17 | 1.56 ± 0.17 | 0.98 ± 0.11 | 0.73 ± 0.08 |
| 221 | α-Amorphene | 1485 | 1482 | 90 | 0.33 ± 0.04 | 0.37 ± 0.04 | 0.39 ± 0.04 | 0.57 ± 0.06 | 0.52 ± 0.06 | 0.26 ± 0.03 |
| 222 | δ-Selinene | 1492 | 1489 | 96 | 0.30 ± 0.03 | 0.49 ± 0.05 | 0.62 ± 0.07 | 0.80 ± 0.09 | 0.35 ± 0.04 | 0.26 ± 0.03 |
| 223 | β-Selinene | 1495 | 1492 | 94 | 0.17 ± 0.02 | 0.31 ± 0.03 | 0.46 ± 0.05 | 0.43 ± 0.05 | 0.46 ± 0.05 | 0.25 ± 0.03 |
| 224 | ε-Amorphene | 1498 | 1502 | 91 | 0.09 ± 0.01 | 0.27 ± 0.03 | 0.29 ± 0.03 | 0.33 ± 0.04 | 0.30 ± 0.03 | 0.13 ± 0.01 |
| 225 | (Z)-β-Guaiene | 1499 | 1498 | 90 | 0.19 ± 0.02 | - | 0.41 ± 0.04 | 0.65 ± 0.07 | - | - |
| 226 | α-Muurolene | 1502 | 1497 | 92 | 0.46 ± 0.05 | 0.62 ± 0.07 | 0.49 ± 0.05 | 0.70 ± 0.08 | 0.10 ± 0.01 | 0.03 ± 0.00 |
| 227 | α-Selinene | 1500 | 1501 | 92 | - | - | - | - | 0.93 ± 0.10 | 0.63 ± 0.07 |
| 228 | β-Bisabolene | 1511 | 1511 | 90 | 0.14 ± 0.02 | 0.09 ± 0.01 | - | 0.06 ± 0.01 | 0.17 ± 0.02 | 0.11 ± 0.01 |
| 229 | 2,4-bis(1,1-Dimethylethyl)-phenol | 1512 | 1519 | 91 | 0.04 ± 0.00 | - | 0.01 ± 0.00 | 0.03 ± 0.00 | - | 0.18 ± 0.02 |
| 230 | δ-Cadinene | 1523 | 1518 | 97 | 0.84 ± 0.09 | 0.53 ± 0.06 | 0.98 ± 0.11 | 0.91 ± 0.10 | 0.31 ± 0.03 | 0.35 ± 0.04 |
| 231 | (E)-Calamenene | 1526 | 1527 | 90 | 0.07 ± 0.01 | 0.10 ± 0.01 | 0.30 ± 0.03 | 0.44 ± 0.05 | 0.11 ± 0.01 | 0.10 ± 0.01 |
| 232 | (E)-Cadina-1,4-diene | 1538 | 1536 | 91 | 0.11 ± 0.01 | 0.11 ± 0.01 | 0.02 ± 0.00 | 0.15 ± 0.02 | 0.13 ± 0.01 | 0.03 ± 0.00 |
| 233 | Selina-4(15),7(11)-diene | 1543 | 1540 | 90 | 0.29 ± 0.03 | - | 0.21 ± 0.02 | 0.36 ± 0.04 | 0.10 ± 0.01 | - |
| 234 | α-Calacorene | 1546 | 1547 | 90 | 0.04 ± 0.00 | 0.08 ± 0.01 | 0.06 ± 0.01 | 0.17 ± 0.02 | 0.06 ± 0.01 | 0.05 ± 0.01 |
| 235 | Selina-3,7(11)-diene | 1548 | 1546 | 92 | 0.16 ± 0.02 | 0.05 ± 0.01 | 0.06 ± 0.01 | 0.09 ± 0.01 | 0.16 ± 0.02 | 0.11 ± 0.01 |
| 236 | Italicene epoxide | 1556 | 1546 | 90 | 0.03 ± 0.00 | 0.12 ± 0.01 | 0.08 ± 0.01 | 0.20 ± 0.02 | 0.19 ± 0.02 | 0.09 ± 0.01 |
| 237 | 2-Methyl-, 4-(2,6,6-trimethyl-1-cyclohexen-1-yl)-but-2-enal | 1578 | 1581 | 92 | 0.01 ± 0.00 | - | - | - | 1.14 ± 0.12 | 0.64 ± 0.07 |
| 238 | Caryophyllene oxide | 1590 | 1587 | 90 | 0.28 ± 0.03 | 0.35 ± 0.04 | 0.44 ± 0.05 | 0.39 ± 0.04 | 0.18 ± 0.02 | 0.20 ± 0.02 |
| 239 | (Z)-3-Hexenyl-benzoate | 1593 | 1573 | 90 | 0.21 ± 0.02 | 0.08 ± 0.01 | - | 0.01 ± 0.00 | 0.04 ± 0.00 | 0.06 ± 0.01 |
| 240 | Caryolan-8-ol | 1594 | 1575 | 91 | 0.03 ± 0.00 | 0.11 ± 0.01 | - | 0.02 ± 0.00 | 0.26 ± 0.03 | - |
| 241 | Caryophyllene alcohol | 1595 | 1575 | 95 | 0.05 ± 0.01 | 0.18 ± 0.02 | 0.02 ± 0.00 | 0.08 ± 0.01 | - | 0.13 ± 0.01 |
| 242 | *n*-Hexadecane | 1600 | 1600 | 90 | 0.03 ± 0.00 | 0.14 ± 0.02 | 0.01 ± 0.00 | 0.15 ± 0.02 | 0.22 ± 0.02 | 0.12 ± 0.01 |

| | | | | | | | | | | |
|---|---|---|---|---|---|---|---|---|---|---|
| 243 | Humulene epoxide I | 1602 | 1604 | 90 | 0.04 ± 0.00 | 0.04 ± 0.00 | 0.08 ± 0.01 | 0.21 ± 0.02 | 0.05 ± 0.01 | 0.02 ± 0.00 |
| 244 | n-Tetradecanal | 1612 | 1614 | 92 | 0.02 ± 0.00 | 0.02 ± 0.00 | 0.02 ± 0.00 | 0.05 ± 0.01 | 0.12 ± 0.01 | 0.02 ± 0.00 |
| 245 | Isopropyl-laurate | 1615 | 1625 | 91 | 0.11 ± 0.01 | 0.56 ± 0.06 | 0.03 ± 0.00 | 0.01 ± 0.00 | 0.02 ± 0.00 | 0.03 ± 0.00 |
| 246 | Humulene epoxide II | 1618 | 1613 | 90 | 0.03 ± 0.00 | 0.05 ± 0.01 | 0.04 ± 0.00 | 0.07 ± 0.01 | 0.08 ± 0.01 | 0.01 ± 0.00 |
| 247 | Muurola-4,10(14)-dien-1-beta-ol | 1632 | 1632 | 90 | 0.02 ± 0.00 | 0.02 ± 0.00 | 0.03 ± 0.00 | 0.02 ± 0.00 | 0.08 ± 0.01 | 0.01 ± 0.00 |
| 248 | *allo*-Aromandendrene epoxide | 1640 | 1644 | 90 | - | - | 0.02 ± 0.00 | 0.03 ± 0.00 | 0.04 ± 0.00 | 0.03 ± 0.00 |
| 249 | Humulenol-II | 1644 | 1650 | 90 | 0.08 ± 0.01 | - | 0.07 ± 0.01 | 0.10 ± 0.01 | 0.07 ± 0.01 | 0.03 ± 0.00 |
| 250 | Caryophylla-4(12),8(13)-dien-5-beta-ol | 1645 | 1636 | 93 | 0.07 ± 0.01 | 0.05 ± 0.01 | 0.10 ± 0.01 | 0.20 ± 0.02 | 0.05 ± 0.01 | 0.05 ± 0.01 |
| 251 | 4-Cadinen-10-ol | 1659 | 1659 | 90 | 0.03 ± 0.00 | 0.07 ± 0.01 | 0.01 ± 0.00 | 0.04 ± 0.00 | 0.04 ± 0.00 | |
| 252 | Germacra-4(15),5,10(14)-trien-1-alpha-ol | 1665 | 1683 | 90 | 0.05 ± 0.01 | - | 0.09 ± 0.01 | 0.11 ± 0.01 | 0.16 ± 0.02 | 0.09 ± 0.01 |
| 253 | Cadalene | 1681 | 1677 | 90 | 0.03 ± 0.00 | 0.05 ± 0.01 | 0.01 ± 0.00 | 0.10 ± 0.01 | 0.17 ± 0.02 | 0.01 ± 0.00 |
| 254 | *n*-Heptadecane | 1700 | 1700 | 94 | 0.01 ± 0.00 | - | 0.02 ± 0.00 | 0.04 ± 0.00 | 0.04 ± 0.00 | 0.07 ± 0.01 |
| 255 | Phytone | 1843 | 1841 | 92 | 0.02 ± 0.00 | 0.01 ± 0.00 | - | 0.03 ± 0.00 | 0.04 ± 0.00 | 0.01 ± 0.00 |
| 256 | (5E,9E)-Farnesyl acetone | 1916 | 1915 | 90 | 0.01 ± 0.00 | 0.02 ± 0.00 | 0.01 ± 0.00 | 0.04 ± 0.00 | 0.01 ± 0.00 | 0.01 ± 0.00 |
| 257 | Methyl-hexadecanoate | 1923 | 1925 | 90 | 0.01 ± 0.00 | - | 0.01 ± 0.00 | 0.05 ± 0.01 | 0.04 ± 0.00 | tr |
| 258 | Cembrene | 1934 | 1939 | 94 | 0.19 ± 0.02 | 0.49 ± 0.05 | 0.17 ± 0.02 | 1.21 ± 0.13 | 0.96 ± 0.10 | 0.91 ± 0.10 |
| 259 | Cembrene ISOMER | 1948 | - | 90 | 0.01 ± 0.00 | - | - | 0.03 ± 0.00 | 0.03 ± 0.00 | 0.02 ± 0.00 |
| 260 | Neocembrene | 1965 | 1960 | 95 | 0.02 ± 0.00 | 0.03 ± 0.00 | 0.01 ± 0.00 | 0.07 ± 0.01 | 0.06 ± 0.01 | 0.05 ± 0.01 |
| 261 | Ethyl palmitate | 1990 | 1993 | 90 | 0.02 ± 0.00 | - | - | 0.02 ± 0.00 | 0.04 ± 0.00 | tr |
| 262 | Cembrene C | 2019 | 2020 | 90 | 0.06 ± 0.01 | - | - | - | 0.02 ± 0.00 | 0.01 ± 0.00 |
| | ***TOTAL*** | | | | **87.49 ± 9.37** | **96.19 ± 10.30** | **86.71 ± 9.29** | **99.47 ± 10.66** | **90.17 ± 9.66** | **97.20 ± 10.41** |

The compounds number is reported in order of elution, considering the total number of compounds eluted. tr-traces

**Table S2.** Identification of the polyphenolic compounds in *R. coriaria* extracts by using HILIC×RP-LC-PDA/MS in positive and negative ionization mode.

| Peak No. | Compound | Chemical family | $T_{tr}$ (min) RSD (%) (n=6) | $[M-H]^-$ /$[M+H]^+$ | $\lambda_{max}$ (nm) | Sample 1 | Sample 2 | Sample 3 | Sample 4 | Sample 5 | Sample 6 |
|---|---|---|---|---|---|---|---|---|---|---|---|
| 1 | Tetragalloyl-hexoside | Gallic acid derivate | 1.515 (0.57) | 787/- | 277 | x | x | x | x | x | - |
| 2 | Pentagalloyl-hexoside | Gallic acid derivate | 1.61 (0.47) | 939/- | 277 | x | x | x | x | x | - |
| 3 | Hexagalloyl-hexoside | Gallic acid derivate | 1.64 (0.66) | 1091/- | 278 | x | x | x | x | x | x |
| 4 | Heptagalloyl-hexoside | Gallic acid derivate | 1.70 (0.75) | 1243/- | 276 | x | x | x | x | x | x |
| 5 | Octagalloyl-hexoside | Gallic acid derivate | 1.73 (0.73) | 1395/- | 276 | x | x | x | x | x | x |
| 6 | Nonagalloyl-hexoside | Gallic acid derivate | 1.78 (0.60) | 1547/- | 275 | x | x | x | x | x | x |
| 7 | Galloyl-valoneic acid bilactone I | Gallic acid derivate | 5.64 (0.04) | 621/- | 279 | - | - | x | x | - | - |
| 8 | Galloyl-valoneic acid bilactone II | Gallic acid derivate | 7.64 (0.06) | 621/- | 278 | - | - | x | x | - | - |
| 9 | Chrysoriol | Luteolin derivate | 11.42 (0.11) | -/301 | 277 | x | x | x | x | x | x |
| 10 | Quercetin rhamnoside I | Quercetin derivate | 11.65 (0.07) | 447/449 | 254, 352 | x | x | - | - | - | - |
| 11 | Quercetin rhamnoside II | Quercetin derivate | 12.50 (0.17) | 447/449 | 254, 352 | x | x | x | x | - | x |
| 12 | Malic acid | Malic acid derivate | 13.19 (0.07) | 133/- | 237 | x | x | x | x | x | x |
| 13 | Levoglucosan gallate | Gallic acid derivate | 13.75 (0.06) | 313/315 | 286 | x | x | x | - | - | - |
| 14 | Protocatechuic acid hexoside | Protocatechuic acid derivate | 15.47 (0.11) | 315/- | 258 | x | x | x | x | x | x |
| 15 | Rutin | Quercetin derivate | 15.75 | 609/- | 266, 353 | x | - | - | - | - | - |
| 16 | Quercetin hexoside I | Quercetin derivate | 15.76 (0.09) | 463/465 | 259, 350 | x | x | x | x | x | x |
| 17 | Quercetin hexoside II | Quercetin derivate | 15.77 (0.11) | 463/465 | 259, 350 | x | x | x | x | x | x |
| 18 | Quercetin hexoside III | Quercetin derivate | 15.80 (0.10) | 463/465 | 259, 350 | - | x | x | x | - | - |
| 19 | Methyl digallate I | Gallic acid derivate | 15.82 (0.03) | 335/- | 265 | x | x | x | x | - | x |
| 20 | Methyl digallate II | Gallic acid derivate | 15.83 (0.02) | 335/- | 265 | - | - | x | x | - | - |
| 21 | Quercetin rhamnoside III | Quercetin derivate | 15.84 (0.11) | 447/449 | 259, 350 | x | x | x | x | x | x |
| 22 | Apiin | Quercetin derivate | 15.88 (0.05) | 563/- | 267, 332 | x | x | x | x | - | - |
| 23 | Quercetin hexoside IV | Quercetin derivate | 15.91 (0.09) | 463/465 | 259, 350 | x | x | x | x | - | - |
| 24 | Quercetin | Quercetin derivate | 15.92 (0.06) | 301/303 | 259, 350 | x | x | - | - | - | x |
| 25 | Gallic acid | Gallic acid derivate | 16.37 (0.08) | 169/- | 277 | x | x | x | x | x | x |
| 26 | Galloyl shikimic acid I | Gallic acid derivate | 16.42 (0.07) | 325/- | 276 | x | x | x | x | - | - |
| 27 | Gallic acid O-malic acid I | Gallic acid derivate | 16.48 (0.08) | 285/- | 276 | x | x | x | x | x | x |
| 28 | Peonidin O-glucoside I | Cyanidin derivate | 16.65 (0.01) | -/463 | 282, 515 | x | x | x | x | x | - |
| 29 | Myricetin | Quercetin derivate | 16.69 (0.09) | -/319 | 260, 359 | x | x | x | x | x | x |
| 30 | Galloylshikimic acid II | Gallic acid derivate | 17.52 (0.15) | 325/- | 273 | x | - | x | x | x | - |
| 31 | Gallic acid O-malic acid II | Gallic acid derivate | 17.56 (0.13) | 285/- | 276 | x | x | x | x | x | x |
| 32 | Apigenin glucoside | Apigenin derivate | 17.80 | -/433 | 265, 344 | - | - | - | - | x | - |
| 33 | Peonidin O-glucoside II | Cyanidin derivate | 17.85 (0.02) | -/463 | 280, 515 | x | x | x | x | - | x |
| 34 | Myricetin O-rhamnosylglucose | Quercetin derivate | 17.92 (0.11) | -/625 | 262, 357 | - | x | x | x | - | x |
| 35 | Myricetin O-glucuronide I | Quercetin derivate | 17.97 (0.05) | 493/495 | 262, 355 | x | x | - | - | - | - |
| 36 | Quinic acid | Quinic acid derivate | 18.20 | 191/- | 237 | x | x | x | x | x | - |

| | | | | | | | | | | | |
|---|---|---|---|---|---|---|---|---|---|---|---|
| | | | (0.09) | | | | | | | | |
| 37 | Galloylshikimic acid III | Gallic acid derivate | 18.47 | 325/- | 274 | - | - | x | - | - | - |
| 38 | Peonidin O-pentoside | Cyanidin derivate | 18.81 (0.24) | -/433 | 273, 503 | x | x | x | x | - | - |
| 39 | Myricetin O-glucuronide II | Quercetin derivate | 18.93 (0.06) | 493/495 | 261, 355 | x | x | x | x | - | x |
| 40 | Quercetin rhamnoside IV | Quercetin derivate | 19.94 (0.02) | 447/449 | 262, 354 | x | x | - | - | - | - |
| 41 | Di-galloyl hexoside I | Gallic acid derivate | 21.70 (0.05) | 483/- | 275 | x | x | x | x | x | x |
| 42 | Cyanidin O-hexoside I | Cyanidin derivate | 21.73 | -/449 | 279, 517 | - | - | x | - | - | - |
| 43 | O-Methyl cyanidinO(2′′galloyl)-galactoside | Cyanidin derivate | 21.89 | -/615 | 278, 518 | - | - | x | - | - | - |
| 44 | Galloyl hexoside I | Gallic acid derivate | 22.20 (0.11) | 331/- | 275 | x | - | x | - | - | - |
| 45 | Cyanidin O-hexoside II | Cyanidin derivate | 22.22 | -/449 | 274, 516 | - | - | x | - | - | - |
| 46 | Di-galloyl hexoside II | Gallic acid derivate | 22.59 | 483/- | 276 | x | - | - | - | - | - |
| 47 | Di-galloyl hexoside III | Gallic acid derivate | 22.70 (0.08) | 483/- | 276 | x | x | x | x | x | x |
| 48 | O-Methyl-cyanidin O(2′′galloyl)-galactoside II | Cyanidin derivate | 22.90 (0.06) | -/615 | 278, 516 | x | x | x | x | - | x |
| 49 | Galloylpyrogallol | Gallic acid derivate | 23.20 (0.11) | 277/- | 238 | x | x | - | - | - | x |
| 50 | Galloyl hexoside II | Gallic acid derivate | 23.37 (0.02) | 331/- | 275 | x | - | x | x | - | - |
| 51 | O-galloylnorbergenin I | Gallic acid derivate | 23.48 (0.11) | -/467 | 276 | x | - | x | x | - | - |
| 52 | Digalloyl hexoside malic acid I | Gallic acid derivate | 23.58 | 599/- | 276 | - | x | - | - | - | - |
| 53 | Di-galloyl hexoside IV | Gallic acid derivate | 23.63 (0.15) | 483/- | 276 | x | - | x | x | x | - |
| 54 | Cyanidin O-hexoside III | Cyanidin derivate | 23.74 (0.02) | -/449 | 279, 518 | - | x | x | x | - | - |
| 55 | Tri-galloyl-hexoside I | Gallic acid derivate | 23.80 (0.14) | 635/- | 276 | x | x | x | x | - | - |
| 56 | O-Methyl-cyanidin O(2′′galloyl)-galactoside III | Cyanidin derivate | 23.89 | -/615 | 278, 516 | - | - | x | - | - | - |
| 57 | Galloyl hexoside III | Gallic acid derivate | 24.21 (0.01) | 331/- | 275 | x | - | x | x | x | - |
| 58 | Di-galloyl hexoside V | Gallic acid derivate | 24.30 (0.01) | 483/- | 274 | - | - | x | x | x | - |
| 59 | O-galloylnorbergenin II | Gallic acid derivate | 25.44 (0.24) | -/467 | 277 | x | - | - | x | - | - |
| 60 | Digalloyl hexoside malic acid II | Gallic acid derivate | 25.52 (0.13) | 599/- | 277 | x | x | x | x | - | - |
| 61 | Trigalloyllevoglucosan I | Gallic acid derivate | 25.67 (0.10) | -/619 | 278 | x | x | x | x | - | x |
| 62 | Digalloyl hexoside malic acid III | Gallic acid derivate | 26.48 (0.12) | 599/- | 277 | x | x | x | x | - | - |
| 63 | Digalloyl hexoside VI | Gallic acid derivate | 26.60 (0.16) | 483/- | 274 | x | x | x | x | x | x |
| 64 | Tri-galloyl-hexoside II | Gallic acid derivate | 26.80 (0.08) | 635/- | 276 | x | x | x | x | - | x |
| 65 | O-galloylnorbergenin III | Gallic acid derivate | 27.43 (0.11) | -/467 | 277 | x | x | x | x | - | x |
| 66 | O-galloylnorbergenin IV | Gallic acid derivate | 27.65 (0.13) | -/467 | 277 | x | - | x | x | - | x |
| 67 | Tri-galloyl-hexoside III | Gallic acid derivate | 27.83 (0.11) | 635/- | 276 | x | x | x | x | - | x |
| 68 | Di-O-galloyl-hexahydroxydiphenoyl-scyllo-quercitol I | Gallic acid derivate | 27.95 (0.10) | -/771 | 278 | x | - | - | - | - | x |
| 69 | Digalloyl hexoside VII | Gallic acid derivate | 28.62 (0.13) | 483/- | 275 | x | x | x | x | - | - |
| 70 | Tri-galloyl-hexoside IV | Gallic acid derivate | 29.73 (0.14) | 635/- | 276 | x | - | x | x | - | - |
| 71 | Di-O-galloyl-hexahydroxydiphenoyl-scyllo-quercitol II | Gallic acid derivate | 29.91 (0.04) | -/771 | 278 | - | x | x | x | - | - |
| 72 | O-galloylnorbergenin V | Gallic acid derivate | 30.62 (0.12) | -/467 | 275 | x | x | x | x | x | x |
| 73 | Tri-galloyl-hexoside V | Gallic acid derivate | 31.80 | 635/- | 276 | x | - | - | - | - | - |

| | | | | | | | | | | | |
|---|---|---|---|---|---|---|---|---|---|---|---|
| | | | (0.02) | | | | | | | | |
| 74 | Cyanidin O-(2''-galloyl) galactoside | Cyanidin derivate | 31.85 (0.05) | -/601 | 279, 517 | x | - | x | x | - | - |
| 75 | Tetra-O-galloylhexoside | Gallic acid derivate | 31.89 (0.01) | 787/- | 277 | - | - | x | x | - | - |
| 76 | O-galloylnorbergenin VI | Gallic acid derivate | 32.58 (0.06) | -/467 | 276 | - | - | x | x | - | - |
| 77 | Trigalloyllevoglucosan II | Gallic acid derivate | 32.63 (0.05) | -/619 | 276 | - | - | x | x | - | - |
| 78 | Tri-galloyl-hexoside VI | Gallic acid derivate | 32.75 (0.03) | 635/- | 276 | x | x | x | - | - | x |
| 79 | Trigalloyllevoglucosan III | Gallic acid derivate | 33.73 (0.04) | -/619 | 276 | - | - | x | x | - | - |
| 80 | Trigalloyllevoglucosan IV | Gallic acid derivate | 34.74 (0.13) | -/619 | 276 | x | - | x | - | - | - |
| 81 | Tri-galloyl-hexoside VII | Gallic acid derivate | 35.68 (0.08) | 635/- | 276 | x | x | x | x | x | x |
| 82 | Tri-galloyl-hexoside VIII | Gallic acid derivate | 35.72 (0.05) | 635/- | 276 | - | - | x | x | - | - |
| 83 | Di-O-galloyl-hexahydroxydiphenoyl-scyllo-quercitol III | Gallic acid derivate | 38.83 (0.09) | -/771 | 278 | x | x | x | x | - | - |
